# Brain and Ventricle Volume Alterations in Idiopathic Normal Pressure Hydrocephalus Determined by Artificial Intelligence-Based MRI Volumetry

**DOI:** 10.3390/diagnostics14131422

**Published:** 2024-07-03

**Authors:** Zeynep Bendella, Veronika Purrer, Robert Haase, Stefan Zülow, Christine Kindler, Valerie Borger, Mohammed Banat, Franziska Dorn, Ullrich Wüllner, Alexander Radbruch, Frederic Carsten Schmeel

**Affiliations:** 1 Department of Neuroradiology, Faculty of Medicine, University Hospital Bonn, Rheinische Friedrich-Wilhelms-Universität Bonn, 53127 Bonn, Germany; zeynep.bendella@ukbonn.de (Z.B.); robert.haase@ukbonn.de (R.H.); stefan.zuelow@ukbonn.de (S.Z.); franziska.dorn@ukbonn.de (F.D.); alexander.radbruch@ukbonn.de (A.R.); 2 German Center of Neurodegenerative Diseases (DZNE), 53127 Bonn, Germany; veronika.purrer@ukbonn.de (V.P.); christine.kindler@ukbonn.de (C.K.); ullrich.wuellner@ukbonn.de (U.W.); 3 Department of Neurodegenerative Diseases, University Hospital Bonn, Rheinische Friedrich-Wilhelms-Universität Bonn, 53127 Bonn, Germany; 4 Department of Neurosurgery, University Hospital Bonn, Rheinische Friedrich-Wilhelms-Universität Bonn, 53127 Bonn, Germany; valerie.borger@ukbonn.de (V.B.); mohammed.banat@ukbonn.de (M.B.)

**Keywords:** normal pressure hydrocephalus, brain atrophy, quantitative neuroimaging, automated volumetrization

## Abstract

The aim of this study was to employ artificial intelligence (AI)-based magnetic resonance imaging (MRI) brain volumetry to potentially distinguish between idiopathic normal pressure hydrocephalus (iNPH), Alzheimer’s disease (AD), and age- and sex-matched healthy controls (CG) by evaluating cortical, subcortical, and ventricular volumes. Additionally, correlations between the measured brain and ventricle volumes and two established semi-quantitative radiologic markers for iNPH were examined. An IRB-approved retrospective analysis was conducted on 123 age- and sex-matched subjects (41 iNPH, 41 AD, and 41 controls), with all of the iNPH patients undergoing routine clinical brain MRI prior to ventriculoperitoneal shunt implantation. Automated AI-based determination of different cortical and subcortical brain and ventricular volumes in mL, as well as calculation of population-based normalized percentiles according to an embedded database, was performed; the CE-certified software mdbrain v4.4.1 or above was used with a standardized T1-weighted 3D magnetization-prepared rapid gradient echo (MPRAGE) sequence. Measured brain volumes and percentiles were analyzed for between-group differences and correlated with semi-quantitative measurements of the Evans’ index and corpus callosal angle: iNPH patients exhibited ventricular enlargement and changes in gray and white matter compared to AD patients and controls, with the most significant differences observed in total ventricular volume (+67%) and the lateral (+68%), third (+38%), and fourth (+31%) ventricles compared to controls. Global ventriculomegaly and marked white matter reduction with concomitant preservation of gray matter compared to AD and CG were characteristic of iNPH, whereas global and frontoparietally accentuated gray matter reductions were characteristic of AD. Evans’ index and corpus callosal angle differed significantly between the three groups and moderately correlated with the lateral ventricular volumes in iNPH patients [Evans’ index (r > 0.83, *p* ≤ 0.001), corpus callosal angle (r < −0.74, *p* ≤ 0.001)]. AI-based MRI volumetry in iNPH patients revealed global ventricular enlargement and focal brain atrophy, which, in contrast to healthy controls and AD patients, primarily involved the supratentorial white matter and was marked temporomesially and in the midbrain, while largely preserving gray matter. Integrating AI volumetry in conjunction with traditional radiologic measures could enhance iNPH identification and differentiation, potentially improving patient management and therapy response assessment.

## 1. Introduction

Diagnosing and monitoring neurodegenerative diseases often involve assessing volume loss in one or multiple brain regions through brain imaging studies. Beyond traditional methods like manual volume measurements through classical segmentation or employing scoring systems, machine learning advancements have led to a rise in automated software solutions available to radiologists. One such solution is artificial intelligence (AI)-driven software for automated brain volumetry for patients suspected of having neurodegenerative diseases, probably complementing standard diagnostic procedures.

Idiopathic normal pressure hydrocephalus (iNPH) poses diagnostic and therapeutic challenges in the realm of neurodegenerative disorders. Characterized by gait disturbance, cognitive decline, and urinary incontinence, iNPH presents with seemingly paradoxical cerebral ventricular enlargement despite normal cerebrospinal fluid (CSF) pressure [1]. Predominantly affecting the elderly, iNPH is often misdiagnosed due to symptom overlap with age-related conditions like Alzheimer’s disease (AD), Parkinson’s disease, or age-related cognitive decline [2]. It is estimated that 1.6–5.4% of individuals with dementia are impacted by iNPH [3]. In fact, iNPH is frequently misidentified as Alzheimer’s disease (AD) due to shared pathophysiological features and comparable imaging characteristics, such as ventricular enlargement and increased white matter lesions (WMLs). The extent to which any patient exhibits reversible symptoms largely depends on the clinician’s ability to accurately differentiate iNPH from other neurodegenerative diseases, such as AD. In cases of severe iNPH, there is a 75% likelihood of overlapping characteristics with AD, complicating the differential diagnosis, especially with standard imaging techniques, and leading to varied outcomes in patient management [4,5]. While several reviews focus on the use of various MRI techniques to detect AD and iNPH separately and assess their clinical utility, almost none comprehensively address the advantages and disadvantages of these techniques in distinguishing between AD and iNPH or their ability to predict shunt responsiveness [6]. It was recently shown that the variations in the extent and distribution of WMLs in iNPH and AD, particularly the predominance of deep WMLs over periventricular lesions in iNPH, were indicative of reduced fluid and amyloid-beta (Aβ) clearance [7]. However, currently, no imaging studies have been able to definitively and reliably diagnose or differentiate between AD and iNPH.

Understanding iNPH pathophysiology necessitates exploring alterations in brain and ventricle volumes. Modern imaging techniques have revealed specific radiological signs aiding iNPH diagnosis and elucidating underlying structural brain changes. Notably, ventriculomegaly in iNPH reflects dynamic CSF flow and cerebral blood flow dynamics, potentially impacting surrounding brain tissue [8]. In the realm of radiological markers, the “Evans’ index” stands out as an emblem of ventriculomegaly, where a ratio greater than 0.3 indicates enlargement of the frontal horns relative to the internal diameter of the skull [9]. Further features include the “tight high convexity”, reflecting narrowing subarachnoid spaces over the brain’s high convexity, and the “disproportionately enlarged subarachnoid-space hydrocephalus (DESH)” pattern, pointing to a combination of ventricular expansion and enlarged Sylvian fissures [10]. Another pivotal sign is the corpus callosum angle. Found at the junction of the corpus callosum and the septum pellucidum in sagittal MRI scans, a diminished or flattened angle in iNPH patients suggests ventricle enlargement’s effects on neighboring cerebral structures [11]. Ishii et al.‘s research emphasizes the angle’s clinical relevance, asserting its capacity to differentiate iNPH from other neurodegenerative conditions [12]. In clinical practice, however, it is often necessary if not mandatory, according to recent guideline recommendations, to combine several of the above-mentioned radiological markers in order to achieve an acceptably high accuracy for the diagnosis of iNPH. This emphasizes the importance and complexity of comprehensive imaging analysis for the accurate diagnosis of iNPH.

Yet, no dedicated quantitative analysis of brain and ventricular volume differences has been conducted for iNPH, as imaging studies have focused primarily on visually assessable and semi-quantitative features. Consequently, it remains unclear whether structural brain changes exist alongside apparent ventricular enlargement and if the quantitative extent of volumetric differences in brain and ventricular size in iNPH can be adequately assessed with currently applied semi-quantitative methods. Objective quantitative imaging markers, together with clinical presentation, could provide a holistic view and pave the way for targeted therapeutic interventions and improved patient outcomes. Therefore, this exploratory retrospective study aims to investigate brain and ventricular volume distributions in clinically confirmed iNPH patients compared to those with AD and age- and sex-matched healthy controls, using AI-based brain magnetic resonance imaging (MRI) volumetry to analyze different cortical, subcortical, and ventricular volumes and to compare these findings with two established semi-quantitative measurement techniques commonly employed in clinical settings.

## 2. Materials and Methods

### 2.1. Study Population

The research received approval from the Ethics Committee for Clinical Trials on Humans and Epidemiological Research with Personal Data at the Faculty of Medicine of the Rheinische Friedrich-Wilhelms-Universität Bonn (reference No. 118/22). Due to the retrospective nature of this study, written informed consent was not required. Adult patients who underwent brain MR imaging in our department in 2000 or later and met the specified inclusion criteria were identified and included in this study through a review of our internal radiologic information system.

The criteria for inclusion encompassed (a) brain MR imaging that featured an unenhanced, three-dimensional (3D), T1-weighted (T1w) sequence; (b) a verified clinical diagnosis of idiopathic normal pressure hydrocephalus (iNPH) with benefit of firstly a corticospinal fluid (CSF) tap test and secondly a response to a following ventricular shunting; and (c) documentation of imaging and clinical pathology related to iNPH diagnosis in the written report. The clinical diagnosis of iNPH was based on the following criteria:

1. A record of walking difficulties, advancing cognitive decline, and a sense of urgency or loss of control over urination;
2. Imaging characteristics of hydrocephalus with disproportionated enlargement of the ventricles in comparison to the outer subarachnoid spaces, whereby an Evans’ index >0.30 was indicative of iNPH on computed tomography (CT) or magnetic resonance (MR) imaging;
3. A CSF opening pressure (appropriately measured) of <24 cm of water.

The Evans’ index is calculated as the ratio between the maximum width of the frontal horns of the lateral ventricles and the greatest internal diameter of the skull on the same axial plane, as observed in CT and MRI images. The CSF tap test involves the assessment of walking and cognitive functions both prior to and following the removal of 40–50 mL of lumbar CSF. It is the sole technique capable of momentarily replicating the impact of a permanent shunt, probably allowing it to forecast not just the results of the surgical procedure but also the extent of recovery [13,14]. 

The International Guidelines have recommended the following key imaging features for the diagnosis of iNPH and the selection of shunt-responsive patients [15,16]:

1. Expansion of the ventricles that cannot be fully ascribed to cerebral shrinkage or congenital enlargement (Evans’ index > 0.3).
2. Absence of any visible blockage impeding the flow of CSF.
3. Presence of at least one of the following indicative characteristics:
  - Enlargement of the temporal horns of the lateral ventricles not entirely attributable to hippocampus atrophy;
  - Callosal angle of <90°;
  - Evidence of altered brain water content, including periventricular signal changes on CT and MRI not attributable to microvascular ischemic changes or demyelination;
  - An aqueductal or fourth ventricular flow void on MRI.

Forty-one patients met these criteria and were grouped into the iNPH cohort. An additional group of 41 patients with clinically diagnosed Alzheimer’s disease (AD) and a healthy control group of 41 patients, with each group being matched for age and gender, were added for group comparisons. The control group was formed from patients who had undergone elective brain MRI for assessment of non-specific headache but exhibited entirely normal brain MR imaging results according to written clinical reports of board-certified radiologists. The general demographic characteristics of the patients are detailed in Table 1.

### 2.2. Magnetic Resonance Imaging

All iNPH patients, AD patients, and healthy controls underwent a standardized brain MR imaging study performed at a clinical 1.5 T or 3 T clinical whole-body MRI system (both Achieva TX, Philips Healthcare, Best, the Netherlands), each equipped with an 8-channel head coil and consistent scanning protocols. Exemplary protocol details regarding the MRI scanning parameters for the 3 T MRI scanner are provided in Table 2. 

### 2.3. Image Analysis

**Figure 1:**
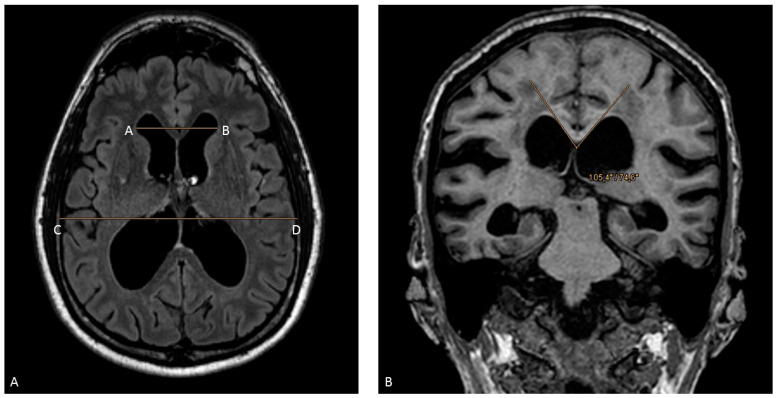
Manual measurements of the Evans’ index: (**A**) The ratio between the maximum width of the frontal horns of the lateral ventricles (line from A to B) and the greatest internal diameter of the skull (line from C to D) on the same axial plane, in an MRI image. The corpus callosal angle (**B**) measured on a coronal image perpendicular to the anterior commissure–posterior commissure (AC-PC) plane at the level of the posterior commissure.

Board-certified radiologists with >5 years of neuroradiological experience visually examined the MRI datasets of all subjects for acute cerebral pathology and the presence of the imaging inclusion criteria. An experienced neuroradiologist (Z.B. with nine years’ experience in neuroimaging) verified all the documented qualitative radiological signs of iNPH and measured two semi-quantitative scores, namely the Evans’ index and the corpus callosal angle. The interpretation of the Evans’ index is as follows: 0.20–0.25, normal; 0.25–0.30, possible or early ventriculomegaly; and >0.30, ventriculomegaly [17]. The ratio varies with age and sex; therefore, the ratio can be higher in a normal healthy elderly individual [18]. The corpus callosal angle should be measured on a coronal image perpendicular to the anterior commissure–posterior commissure (AC-PC) plane at the level of the posterior commissure [16,19]. INPH patients have smaller angles than those with ventriculomegaly from atrophy or normal controls: a normal value is between 100 and 120°; in patients with iNPH, the value is <90°. Examples of both radiological markers are visually shown in Figure 1. 

### 2.4. Post-Processing and Artificial Intelligence (AI)-Based Volumetry

An automated AI-driven software tool was employed to perform quantitative volume analyses of various brain regions, measured in mL. The analysis was supplemented by age- and gender-adjusted percentiles derived from an internal reference group of age- and gender-matched healthy individuals embedded in the software. This MRI post-processing software, “mdbrain”, is a product of Mediaire GmbH, Berlin, Germany, a certified manufacturer under the European Medical Device Directive 93/42/EEC, and also aligns with the DIN EN ISO 13485:2016 standards [20]. The “mdbrain” software, holding a CE mark, facilitates automatic brain volumetry of numerous brain sections using 3D T1-weighted sequences, leveraging a vast normative database from the general population. Importantly, this software’s algorithm and its encompassed normative database are refined based on a broad dataset of multi-center imaging data, surpassing the confines of a single institution, and was endorsed for its precision [21,22].

**Figure 2:**
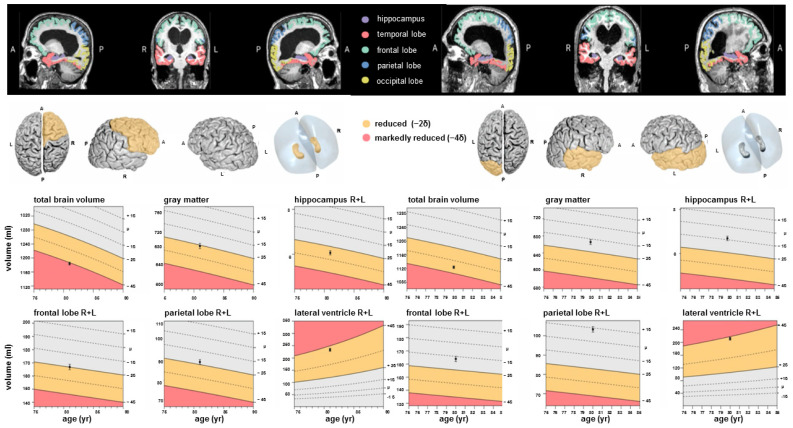
Sample excerpt of the software output (headings modified by the authors for translation from German) of two exemplary patients. The black dot within the percentile image indicates the patient’s individual percentile in the corresponding brain area. Abbreviations: A = anterior, P = posterior, R = right and L = left. It is noteworthy that volume measurements for symmetrical structures were conducted individually for each counterpart. The analyzed cortical and subcortical brain structures and ventricles included the following: whole brain volume, whole brain white matter, whole brain gray matter, cortical gray matter, frontal volume right and left, parietal volume right and left, precuneus volume right and left, occipital volume right and left, temporal volume right and left, hippocampus volume right and left, parahippocampal gyrus volume right and left, regio entorhinalis volume right and left, nucleus caudatus volume right and left, putamen volume right and left, pallidum volume right and left, thalamus volume right and left, brainstem volume, mesencephalon volume, pons volume, cerebellar gray matter volume, left and right ventricle, third and fourth ventricle.

The 3D T1w MPRAGE sequence was uploaded from the clinical PACS into the mdbrain software, version 4.4.1 or subsequent versions, to facilitate automatic volume determination and percentile assessment. Afterward, volumes and percentiles for a comprehensive list of structures—from the entire brain to individual ventricles—were automatically generated, stored, and then verified for their credibility, as shown in Figure 2. 

The automated volumetry process includes the following steps [23,24]:

1. Segmenting the structures of interest. A custom deep learning segmentation model, based on the 3D U-Net architecture [25], is utilized for this purpose. Input to the model is a 3D T1 MRI scan (cropped to include only the head and resampled to a uniform size), and the output of the model is a 3D segmentation mask, containing segmentation of various brain structures as listed above. The model was trained using the Adam optimization algorithm, a variant of stochastic gradient descent. The training dataset consisted of 2869 gender-balanced MRI scans and corresponding segmentation annotations created through a proprietary multi-rater annotation process. Various augmentation techniques, such as contrast, resolution, rotation, and elastic deformation adjustments, were applied during training to enhance the model’s generalizability. The algorithm was validated on a test dataset consisting of MRI scans from 121 subjects and the corresponding manual segmentations by human experts.
2. Calculating the volume of the segmented structures by counting the number of voxels in the segmentation mask and multiplying by the voxel volume.
3. Comparing the calculated volumes to a reference population of healthy individuals (n = 6099, balanced for gender, mean age 41 ± 23 years, age range 10–97 years, with diverse image sources from Europe, the United States, Australia, and China) to determine percentiles, accounting for age, sex, and total intracranial volume.

As per the instructions for use of mdbrain, the AI software has the following limitations:

- Use is restricted to certain allowed MR scan parameter ranges (for example, the spacing between slices must be below 2 mm);
- Quality of results cannot be guaranteed for patients with an age outside the range of 10–99 years or those with tumor or stroke;
- Comparability of results is not guaranteed if the image data were recorded with different MR sequences.

### 2.5. Statistical Analysis

Statistical analyses were conducted using SPSS statistical software (v27 and above, IBM Corp., Armonk, NY, USA). All applicable demographic and imaging data are given as mean ± standard deviation, unless otherwise specified. The statistical significance level was set at *p* = 0.05. Mann–Whitney U and Kruskal–Wallis tests were used for pairwise and multiple group comparisons of independent clinical and imaging data, and one-way ANOVA with post hoc testing after Bonferroni correction was used for pairwise inter-class comparisons. Spearman’s rank-order correlation was used to assess for potential associations between the measured Evans’ index and corpus callosal angle with the AI-based volumetrically measured total ventricle volume. Receiver operating characteristic (ROC) curves were plotted to determine the optimal Evans’ index, corpus callosal angle, and total ventricle volume thresholds in order to differentiate iNPH from healthy controls and AD patients. The sensitivity, specificity, and diagnostic accuracy of both semi-quantitative and quantitative imaging parameters were calculated using these cut-off values.

## 3. Results

The general patients’ demographics including age and gender distribution showed no statistically significant differences among the three groups of iNPH, AD, and healthy controls (CG), as shown in Table 1.

### 3.1. Brain Volume Deviations in iNPH Patients as Compared to a Norm Collective

**Figure 3:**
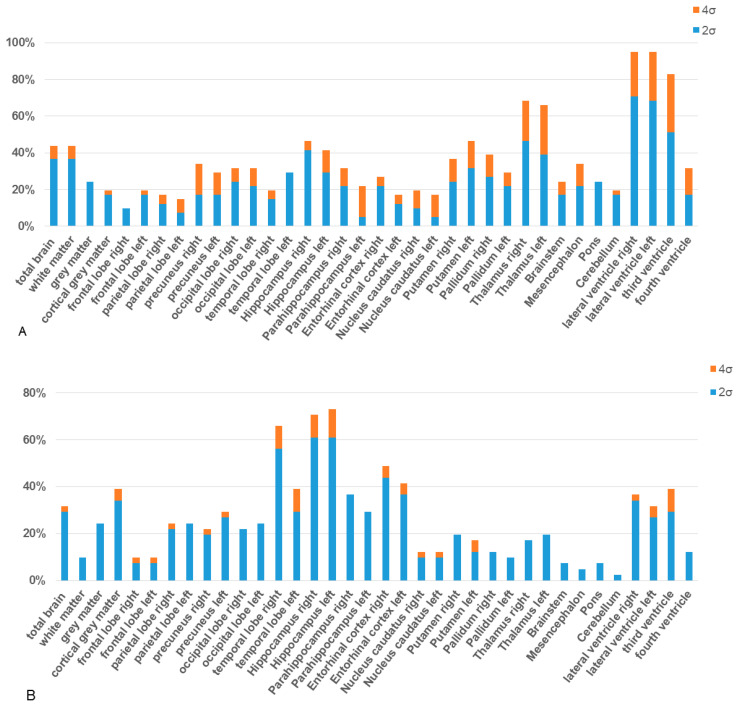
Proportion of subjects with volume deviations among iNPH patients (**A**) and AD patients (**B**) is presented. The incidence of these deviations, either by 2 (illustrated in blue) or 4 (in orange) standard deviations, is compared with a healthy, population-based control group embedded in the AI-based volumetric software. The deviations of the supratentorial volumes are provided separately for each side of the brain. Deviations in cortical and subcortical volumes indicate atrophy, whereas deviations in ventricular volumes suggest enlargement.

In a comparison of the individual brain (sub)volumes of iNPH patients with the embedded population-based norm collective, a significant proportion of subjects with volume deviations from the norm collective was found, especially in the lateral ventricles and third ventricle, both thalami, hippocampi, and putamina, as detailed in Figure 3 (Supplementary Table S1).

### 3.2. Artificial Intelligence-Based Volumetric Results

The volumetry software successfully processed all MR imaging studies, and the measurement results of brain area and ventricle volumes in mL and the corresponding relative measurement values normalized to the total intracranial volume (ICV, sum of total brain volume and total ventricle volume) for the three participant groups, iNPH, AD, and controls, are shown in Table 3 (absolute volume measurements in mL) and Table 4 (relative volumes normalized to ICV). In the comparative analysis of brain volumetric measurements in mL among groups, several significant differences were observed across various brain regions: Overall, ICV in mL was significantly higher in iNPH patients compared to both controls and AD groups, with mean ICV values of 1231.81 ± 144, 1181.17 ± 98, and 1115.96 ± 115, respectively (*p* < 0.001). These differences represented relative volume increases of 4% compared to controls and 10% compared to the AD patients. Regarding total brain volume, iNPH patients exhibited lower volumes compared to controls but higher volumes compared to AD patients, with mean mL values of 1102.46 ± 122, 1138.41 ± 93, and 1050.72 ± 110, respectively (*p* = 0.003). The relative volume reduction was 3% for iNPH compared to controls, and the relative volume increase was 5% compared to AD patients. In terms of specific brain regions, notable differences were observed in gray matter and white matter volumes in mL: iNPH patients displayed a mean gray matter volume of 638.69 ± 64 and a mean white matter volume of 462.07 ± 83. These volumes were lower compared to controls (gray matter: 641.54 ± 55; white matter: 496.87 ± 46), higher compared to AD gray matter (576.48 ± 59), and lower compared to AD white matter (474.24 ± 56). The relative volume increases in gray matter were 0% compared to controls and 10% compared to AD patients, while in white matter, volume reductions were 8% compared to controls and 3% compared to AD patients. Significant differences were also found in specific brain regions such as the frontal lobe, parietal lobe, precuneus, occipital lobe, temporal lobe, hippocampus, parahippocampus, entorhinal lobe, caudate, putamen, pallidum, thalamus, brainstem, mesencephalon, pons, and cerebellum among the three groups. For instance, in the temporal lobe, iNPH patients exhibited a mean mL volume of 116.03 ± 16, which was lower than that of controls (130.11 ± 12) and higher than that of AD patients (103.79 ± 14). This corresponded to a relative volume reduction of 12% compared to controls and to an increase of 11% compared to AD patients (*p* < 0.001). In the frontal lobe, iNPH patients exhibited a 1% reduction compared to controls and a 6% increase compared to AD patients. In the hippocampus, iNPH patients had a mean mL volume of 5.77 ± 1, whereas controls and AD patients showed volumes of 6.72 ± 1 and 5.79 ± 4, respectively. This indicated a relative volume reduction of 14% compared to controls and no difference compared to AD patients (*p* < 0.001).

Regarding ventricular volumes, iNPH patients showed substantial enlargement, with a 67% increase in total ventricular volume compared to controls. Specifically, the lateral ventricles displayed a 68% increase in volume, with percentage changes of 69% for the right lateral ventricle and 67% for the left lateral ventricle, while the third and fourth ventricles exhibited percentage changes of 38% and 31%, respectively, compared to controls. AD patients also displayed ventricular enlargement, with a 50% increase in total ventricular volume compared to controls. Specifically, the lateral ventricles exhibited a 50% increase in volume, with percentage changes of 50% for the right lateral ventricle and 51% for the left lateral ventricle, while the third and fourth ventricles demonstrated percentage changes of 28% and 29%, respectively, compared to controls.

In the relative measurements normalized to ICV, the most prominent differences between the groups are seen in the relative volumes of mesial structures such as the hippocampus and the parahippocampus. The relative volume of the hippocampus in iNPH is 0.0050, while it is 0.0057 in CG and 0.0053 in AD. Similarly, the relative volume of the parahippocampus is 0.0041 in iNPH compared to CG (0.0055) and AD (0.0049). These differences correspond to percentage changes of −17% and −8% for the hippocampus and −34% and −20% for the parahippocampus in iNPH compared to CG and AD.

Additional subgroup analysis revealed significant differences in various brain regions and ventricular volumes among patients with iNPH, AD, and controls (Table 5 and Table 6). Notable differences were observed in total ventricular volume, lateral ventricular volume, and the volumes of the third and fourth ventricles, with significant mean differences found between iNPH and both other groups (controls and AD) (*p* < 0.001). The mean differences for total ventricle volume between iNPH and CG, iNPH and AD, and controls and AD were 86.60, 64.11, and 22.49, respectively, with corresponding standard errors of 7.16. Similarly, for the lateral ventricular volume, the mean differences were 85.05, 62.85, and 22.20, with standard errors of 7.02. Regarding brain volumes, while differences in total brain volume were not statistically significant between iNPH and controls, a borderline significant difference was detected between iNPH and AD groups (mean difference = 51.74, SE = 24.15, *p* = 0.103). White matter volume exhibited significant differences between iNPH and control groups (mean difference = −34.80, SE = 14.04, *p* = 0.044), although no significant differences were observed between iNPH and AD or control and AD groups. Cortical gray matter volume showed a marked discrepancy between iNPH and AD groups (mean difference = 39.21, SE = 11.85, *p* = 0.004), along with significant differences between control and AD groups (mean difference = −59.31, SE = 11.85, *p* < 0.001). Notably, significant distinctions were observed in various lobes, including the frontal, parietal, precuneus, temporal, and hippocampal regions.

### 3.3. Semi-Quantitative Markers for the Diagnosis of iNPH 

**Figure 4:**
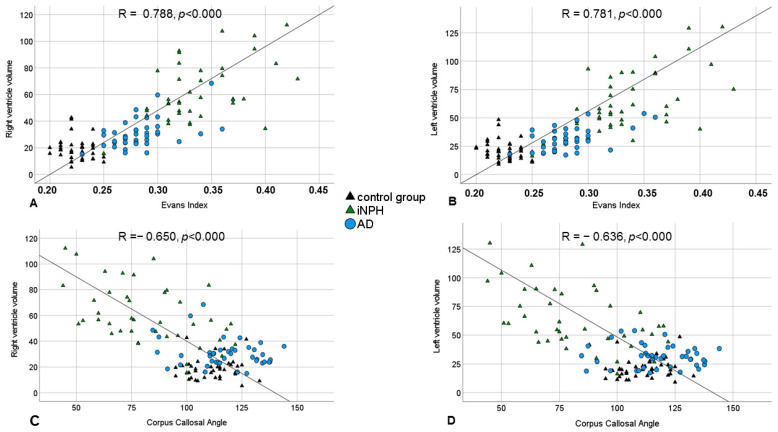
Scatterplot of the right (**A**,**C**) and left (**B**,**D**) ventricle volumes and the values of the Evans’ index (**A**,**B**) and corpus callosum angle (**C**,**D**).

The mean Evans’ index and corpus callosal angle were statistically significantly different between the iNPH, AD, and control groups (Evans’ index: iNPH 0.34 ± 0.04 vs. AD 0.28 ± 0.03 vs. control group 0.23 ± 0.01, *p* ≤ 0.001; corpus callosal angle: iNPH 82.22 ± 22.12 vs. AD 117.60 ± 15.23 vs. control group 111.59 ± 9.59, *p* ≤ 0.001). As graphically illustrated in Figure 4, the total ventricle volume of the iNPH patients and the measured Evans’ index and corpus callosal angle were found to correlate moderately (Evans’ index: right lateral ventricle r = 0.788, *p* = 0.000, and left lateral ventricle r = 0.781, *p* ≤ 0.001; corpus callosal angle: right lateral ventricle r = −0.650, *p* ≤ 0.001, and left lateral ventricle r = −0.636, *p* ≤ 0.001).

**Figure 5:**
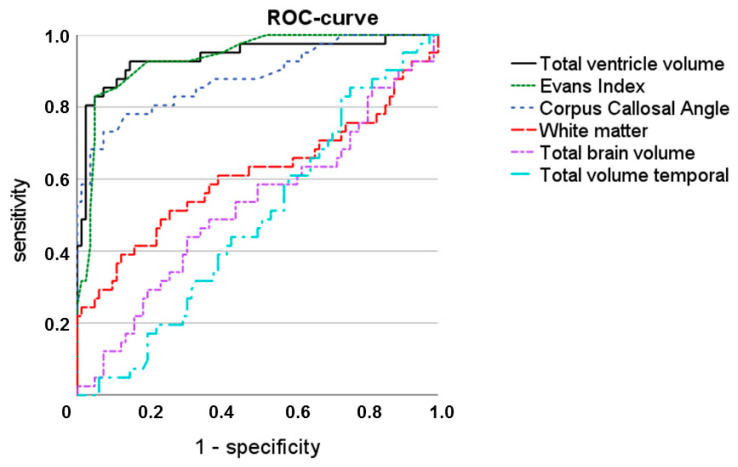
ROC curve with total ventricle volume, Evans’ index, corpus callosum angle, white matter, total brain volume, and total temporal volume.

Both semi-quantitative measurement ratios, the Evans’ index and the corpus callosal angle, and the automatically determined total ventricular volume allowed for a differentiation between the iNPH and healthy control groups with a moderate to high diagnostic accuracy, as shown in the corresponding ROC curves in Figure 5 and further specified in Table 7. 

## 4. Discussion

Idiopathic NPH stands as one of the limited number of curable sources of dementia, requiring a collaborative effort among neurologists, neurosurgeons, and neuroradiologists for accurate diagnosis. Identifying iNPH proves to be challenging due to its inconsistent symptoms and progression and the fact that it often coexists or is confused with other neurological conditions. Therefore, a significant amount of research has been dedicated to creating a reliable, noninvasive predictive test for iNPH.

In this study, we evaluated brain volumes of patients with iNPH compared to those with AD and healthy controls. Using automated, AI-based brain MRI volumetry, we aimed to identify morphometric changes across these groups. This study represents the largest MRI investigation of volumetric analysis in iNPH patients to date, as far as we are aware. Our volumetric analyses unveiled substantial differences in ventricular size and small to moderate, yet statistically significant, volumetric disparities in cortical and subcortical brain regions among iNPH patients compared to those with AD and healthy controls. These differences primarily manifested as global ventriculomegaly alongside concurrent atrophy of the entire white matter, thalamus, brainstem, and midbrain, with notable reductions in brain volume observed particularly in temporomesial and midline structures. These patterns of ventricular enlargement and focal brain atrophy were corroborated by corresponding alterations in normalized percentiles derived from a normal population database integrated into our analysis software, thus confirming the existence of structural brain changes commonly reported in the literature. Notably, iNPH patients exhibited significant enlargement of all ventricles including the third and fourth ventricle alongside reductions in whole brain white matter, midbrain, and mesial structures, while largely preserving the neocortical gray matter in comparison to healthy controls and AD. This contrasts with the significant reduction observed in both the gray and white matter of the cerebrum in AD. These findings align with previous studies and provide quantitative evidence supporting the notion that larger global ventricular size with preservation of neocortical structures is indicative of iNPH.

In our iNPH patients, the volumes of the third and fourth ventricles were significantly enlarged compared to AD patients, while there was no difference in the lateral ventricles among these two entities. Moreover, the whole and supratentorial grey matter volumes were significantly lower in AD than in iNPH patients, with a notable volume reduction of AD patients in the frontal and parietal regions compared to iNPH. In contrast, both groups demonstrated substantial volume reductions in the temporal region. The findings are broadly consistent with the current literature, highlighting a decrease in volume surrounding the expanded ventricles in iNPH patients as CSF studies in patients with iNPH have shown subsequent neuronal degeneration [26]. The pathophysiology underlying these volume changes remains an area of ongoing investigation, with some studies suggesting that the compression exerted by the enlarged ventricles may contribute to brain tissue reduction [27]. Additionally, it points out that AD patients typically exhibit pronounced cortical atrophy, especially in the parietotemporal and frontal regions [28,29,30]. Our findings additionally demonstrated focal brain atrophy of the temporal, precuneus, and frontal lobes in iNPH patients as compared to healthy controls, potentially indicating the cerebrospinal fluid pressure exerted on these brain areas that are thought to probably cause the Hakim triad.

In clinical practice, the assessment of ventricular size and potential structural changes in iNPH typically relies on subjective visual inspection or semi-quantitative measurements, such as the Evans’ index or the corpus callosum angle, in computed tomography (CT) and/or MRI scans [16,17,18,19]. With regard to traditional semi-quantitative parameters, our measurements of the Evans’ index and the corpus callosum angle in iNPH showed moderate correlations with the lateral ventricular width, with the correlation of the corpus callosum angle being slightly weaker than the correlation with the Evans’ index. These observations may suggest that the two aforementioned parameters represent a practical measure for estimating ventricular enlargement. However, visual assessment and semi-quantitative estimation of ventricular volume are prone to error due to sensitivity to the choice and angle of multiplanar image reconstructions, particularly with indices like the Evans’ index and corpus callosal angle. These limitations, combined with individual anatomical variations, can significantly affect the correlation between these semi-quantitative radiological markers and ventricular volume. While recent studies have explored the utility of the Evans’ index in distinguishing individuals with iNPH from healthy subjects [31,32,33,34], its application necessitates strict adherence to specific image reconstruction planes, and its efficacy in post-surgical patient monitoring remains uncertain. Similarly, morphological imaging indicators associated with iNPH, such as a decreased corpus callosum angle and disproportionately enlarged subarachnoid space, are reliant on operator expertise. Although these indicators demonstrated some correlation with ventricular volume and the ventricular volume to intracranial volume (ICV) ratio, the correlation was shown to be not robust: variability in brain CT and MRI protocols across different imaging facilities, including differences in slice thickness and angulations, undermines the reliability of comparing the Evans’ index across scans, even for the same patient [35]. This variability complicates the comparison of pre- and postoperative scans, as well as the comparison of ventricular volumes between iNPH patients and those without iNPH. While this may not greatly affect patients with significantly enlarged ventricles, it holds critical implications for patients with borderline ventricle sizes, both in research on iNPH and in clinical management [35]. The Evans’ index previously demonstrated a high combined sensitivity of 96%, but its specificity was relatively low at 83% in diagnosing iNPH [36]. Similarly, the corpus callosal angle showed a comparable combined sensitivity and specificity of 89% and 92%, respectively [36]. Consequently, while the Evans’ index and the corpus callosum angle are useful in most instances for substantiating a morphologically suspected diagnosis with semi-quantitative parameters, they ultimately do not allow one to reliably diagnose and differentiate iNPH from other neurodegenerative diseases, such as Alzheimer’s dementia, in terms of their combined sensitivity and specificity. 

Advancements in artificial intelligence (AI) have facilitated the development of automated volumetry tools, which have shown promise in providing a more nuanced understanding of iNPH. These AI-driven tools are thought to assess brain volume with high precision and consistency, potentially identifying subtle changes that may be overlooked in visual assessments. By integrating machine learning algorithms with traditional imaging data, it becomes possible to generate more accurate and reliable diagnostic criteria. The advent of AI and machine learning in neuroimaging has added a layer of precision to the assessment of brain volume loss and its relationship with radiological signs in iNPH and may explain the significant brain volume differences observed in our cohort involving gray and white matter, brainstem and midline volume, and ventricular size. The investigator-independent objectivity that can be achieved by an automated volumetry of MR images may hold the potential to substantially improve both diagnosis and follow-up of iNPH patients while adding just a few minutes of automated postprocessing time to the whole examination. To date, some semi-automated volumetric techniques have been effectively employed in research focusing on changes in ventricular volume post-surgery in iNPH [37,38]. It can be expected that fully automated segmentation methods such as the CE-marked AI-based software used in this study will further improve the accuracy and time efficiency of cerebral and ventricular volume measurements in patients with iNPH, including postoperative assessments. According to our volumetric data, even small but statistically significant decreases in cerebral white matter volume in the presence of global ventriculomegaly and largely preserved age-normalized cerebral gray matter are strongly suggestive of the presence of iNPH—findings that, due to their often subtle nature, may elude visual assessment even by an experienced examiner. Knowledge of this focal pattern of structural cerebral alteration, as opposed to the global and particularly frontoparietal accentuated cortical atrophy in AD patients, should potentially enhance diagnostic accuracy. However, larger trials should be conducted to identify specific volumetric cut-off values to eventually substantiate the value of AI volumetrization for diagnosing iNPH. 

We acknowledge several limitations of this study. The retrospective nature generally limits the conclusions to be drawn since the reproducibility of the obtained clinical and imaging data cannot be determined. Also, we are aware of the relatively small sample size. However, we included only those patients who had a standardized, in-house MRI scan before ventriculoperitoneal shunt placement and a positive cerebrospinal fluid opening pressure. Furthermore, all of our AD patients were classified at a lower level (Levels 3 (mild cognitive impairment) and 4 (mild dementia)) according to the criteria of the Diagnostic and Statistical Manual of Mental Disorders, fourth edition (DSM-IV), and of the National Institute of Neurologic and Communicative Disorders and Stroke and the Alzheimer’s Disease and Related Disorders Association [39]. It is, therefore, uncertain whether AD patients in a more advanced stage of the disease show more or less distinct brain structural differences from iNPH. The limiting factor of AI is the “black box” nature of many AI models, particularly deep neural networks, which complicates their integration into clinical decision-making. The lack of transparency in AI’s decision-making processes can be a significant hurdle, as medical professionals rely on clear, understandable reasons behind diagnostic decisions. AI models can also suffer from overfitting, where they excel on training data but perform poorly on new, unseen data, or underfitting, where the model is too simplistic to capture the data’s complexities. However, our software’s algorithm and its encompassed normative database were refined based on a broad dataset of multi-center imaging data, surpassing the confines of a single institution. Lastly, the commercial AI-based mdbrain package used in this study is a proprietary software solution, so reproducibility with other software solutions is not readily possible. In order to determine what the underlying network structure actually looks like and how specific hyperparameters were defined—information that is not available to us in detail—one would have to contact the manufacturer, Mediaire. However, it will be easy to reproduce our results using the commercial software. 

## 5. Conclusions

In conclusion, iNPH patients exhibited distinct brain volume alterations with patterns of global ventriculomegaly and white matter loss in addition to thalamic, brainstem, and temporomesial volume loss, while neocortical gray matter was largely preserved. Our volumetric results are supported by a concomitant decrease in population-normalized percentiles in the corresponding brain areas. These findings could potentially facilitate the radiological differentiation of patients with iNPH from healthy controls and AD, requiring only a few minutes of additional software-based post-processing. While traditional radiologic signs probably remain central to the understanding and diagnosis of iNPH in clinical practice, the inclusion of AI-driven measurements may provide an additional level of precision. Future research should focus on refining and validating volumetric markers and developing a comprehensive approach that aligns clinical and radiologic findings. This would not only improve diagnostic accuracy but also pave the way for more effective therapeutic interventions in iNPH.

## Figures and Tables

**Table 1:** Demographic characteristics of the study participants (n = 82).

	NPH (n = 41)	Control Group (n = 41)	Alzheimer’s Disease (n = 41)	*p*-Value ^†^
**Age—yr**	79.4 ± 7.5	79.0 ± 4.9	81.6 ± 8.4	0.766
**Male sex—No. (%)**	28 (68%)	27 (66%)	21 (51%)	0.817

^†^ a *p*-value < 0.05 was considered statistically significant. Group differences were calculated using the Kruskal–Wallis test.

**Table 2:** Sequence parameters.

Sequence	Pulse Type	Orientation	TR (ms)	TE (ms)	Reconstructed Voxel Size (mm)	Matrix (mm)	Slices
T2w	Turbo spin echo	axial	13.257	90	0.94 × 0.94 × 1	240 × 174	140
SWI	3D fast field echo	axial	31	0	0.6 × 0.6 × 2	384 × 316	145
DWI	*b* values (0, 500, 1000 s/mm^2^)	axial	2725	41	1 × 1 × 5	128 × 127	24
T1w	MPRAGE	sagittal	7.3	3.9	1 × 1 × 1	256 × 256	180
FLAIR	3D gradient echo	sagittal	4800	275.776	1.12 × 1.12 × 1.12	240 × 240	321

DWI, diffusion-weighted imaging; MPRAGE, magnetization-prepared rapid gradient echo; SWI, susceptibility-weighted imaging; T1w, T1-weighted; T2w, T2-weighted; TE, echo time; TR, repetition time.

**Table 3:** Results of volumetric MRI analysis in iNPH and AD patients and the control group, given in absolute volumes [mL].

	iNPH (n = 41)		Control Group (CG) (n = 41)		Alzheimer’s Disease (AD) (n = 41)		iNPH vs. CG	iNPH vs. AD	*p*-Value ^#^
	Mean	SD	Mean	SD	Mean	SD	% Δ	% Δ	
**ICV**	1231.8122	144.1960	1181.1659	97.8317	1115.9610	115.0844	4	10	0.000
**Total brain volume**	1102.4610	122.2758	1138.4146	93.2713	1050.7220	110.5777	−3	5	0.003
**White matter**	462.0683	82.6898	496.8659	45.7924	474.2415	56.4358	−8	−3	0.025
**Gray matter**	638.6902	64.4441	641.5390	54.7634	576.4780	58.6856	0	10	0.000
**Cortical gray matter**	417.1683	46.5077	437.2683	39.6168	377.9537	70.0438	−5	9	0.000
**Frontal lobe**									
Total	155.5732	18.5901	157.3951	15.6656	145.6000	17.3142	−1	6	0.006
Right	79.5854	9.5334	79.6146	8.1767	73.5463	8.7155	0	8	0.002
Left	75.9878	9.2762	77.7805	7.5898	72.0537	8.8034	−3	5	0.018
**Parietal lobe**									
Total	84.8756	12.3904	86.3463	8.0252	78.1098	9.4089	−2	8	0.000
Right	41.8171	6.4713	41.9829	3.9911	38.4707	4.8411	0	8	0.002
Left	43.0585	6.3699	44.3634	4.1813	39.6390	4.9422	−3	8	0.000
**Precuneus lobe**									
Total	17.8976	5.8758	20.9902	2.4871	18.1268	3.0642	−16	0	0.000
Right	8.8927	3.0532	10.2854	1.2165	9.0951	1.5105	−14	0	0.001
Left	9.0049	2.9051	10.7049	1.3405	9.0317	1.6951	−18	0	0.000
**Occipital lobe**									
Total	60.6756	11.5576	63.3610	7.1624	57.9146	8.0108	−5	5	0.004
Right	29.4439	5.8061	30.3220	3.3806	27.9439	4.1954	−3	6	0.013
Left	31.2317	6.1485	33.0390	4.0683	29.9707	4.0727	−7	4	0.006
**Temporal lobe**									
Total	116.0268	15.5967	130.1073	11.7281	103.7902	14.0070	−12	11	0.000
Right	58.7976	9.0286	66.2951	6.2910	52.5171	7.4310	−13	10	0.000
Left	57.2293	7.4943	63.8122	5.7790	51.2732	7.1145	−11	11	0.000
**Hippocampus**									
Total	5.7683	1.0999	6.7171	0.7466	5.7927	3.8273	−14	0	0.000
Right	2.9854	0.5677	3.5683	0.4245	3.3171	3.8189	−29	−14	0.000
Left	2.7829	0.5749	3.1488	0.3565	2.4756	0.4603	−13	12	0.000
**Parahippocampus**									
Total	5.3683	1.4093	6.4756	0.8040	5.4220	0.8027	−21	−1	0.000
Right	2.6146	0.7562	3.1902	0.4364	2.6439	0.4411	−22	0	0.000
Left	2.7537	0.7036	3.2854	0.3997	2.7780	0.3947	−19	−1	0.000
**Entorhinal lobe**									
Total	3.9732	0.9260	4.7585	0.7050	3.4732	0.7934	−20	12	0.000
Right	1.9878	0.5109	2.4220	0.3883	1.7341	0.4059	−23	13	0.000
Left	1.9854	0.4503	2.3366	0.3569	1.7390	0.4254	−19	13	0.000
**Caudate**									
Total	6.0902	2.1805	6.0634	1.2397	5.9049	1.5079	1	3	0.662
Right	3.1878	1.1860	3.1976	0.6436	3.0805	0.8054	0	4	0.534
Left	2.9024	1.0613	2.8659	0.6315	2.8244	0.7599	2	3	0.713
**Putamen**									
Total	7.0854	1.6249	7.8927	0.9863	2.5976	0.3453	−12	64	0.731
Right	3.5707	0.7329	3.9049	0.5059	3.7439	0.5714	−9	−6	0.136
Left	3.5146	0.9372	3.9878	0.5154	3.7878	0.6282	−13	−7	0.069
**Pallidum**									
Total	2.4902	0.4415	2.5512	0.3487	2.5463	0.3805	−2	−2	0.731
Right	1.2098	0.2300	1.2634	0.1959	1.2951	0.1788	−5	−8	0.446
Left	1.2805	0.2283	1.2878	0.1646	1.3024	0.1753	0	−3	0.807
**Thalamus**									
Total	12.7439	2.6397	14.6122	1.1752	15.5951	9.0255	−14	−22	0.000
Right	6.3390	1.3687	7.1244	0.6041	6.9951	0.6753	−12	−10	0.001
Left	6.4049	1.3638	7.4878	0.6133	8.6000	9.0765	−17	−34	0.000
**Brainstem**	25.4146	3.6199	26.2366	2.3313	27.0537	2.6888	−3	−6	0.055
**Mesencephalon**	7.1073	0.9429	7.3390	0.6016	8.1707	3.4995	−3	−15	0.016
**Pons**	13.9805	2.4003	14.9317	3.9517	14.2951	2.5724	−7	−2	0.304
**Cerebellum**	90.5610	11.1429	99.7610	9.8351	94.0366	17.9692	−10	−4	0.001
**Ventricle volume**	129.3512	50.0321	42.7512	16.5050	65.2390	19.4649	67	50	0.000
**Lateral ventricle**									
Total	124.8488	49.0248	39.8000	16.0337	61.9976	19.1541	68	50	0.000
Right	60.6195	23.0739	18.6829	8.0216	30.5244	10.8250	69	50	0.000
Left	64.2293	26.8961	21.1171	8.3129	31.4732	9.7894	67	51	0.000
**Third ventricle**	2.4902	0.7816	1.5390	0.5098	1.8049	0.4779	38	28	0.000
**Fourth ventricle**	2.0122	0.8051	1.4122	0.4008	1.4366	0.3352	31	29	0.000

Results of volumetric MRI analysis in iNPH and AD patients and the control group, given in absolute volumes. Mean absolute volumes [mL] of the different brain volumes for iNPH, control group, and AD patients. ^#^ *p*-value of the Kruskal–Wallis test. *p*-value < 0.005 was considered statistically significant. % Δ percentage difference of the mean of iNPH patients to the mean of healthy patients in the control group (CG) and to the mean of AD patients. Abbreviations: SD = standard deviation. ICV = intracranial volume. iNPH = idiopathic normal pressure hydrocephalus. AD = Alzheimer’s disease. CG = control group.

**Table 4:** Results of volumetric MRI analysis in iNPH and AD patients and the control group in relative volumes, normalized to the total intracranial volume (ICV) of each subgroup.

	iNPH (n = 41)		Control Group (CG) (n = 41)		Alzheimer’s Disease (AD) (n = 41)		iNPH vs. CG	iNPH vs. AD	*p*-Value ^#^
	Mean	SD	Mean	SD	Mean	SD	% Δ	% Δ	
**ICV**	1.000		1.000		1.000				
**Total brain volume**	0.8961	0.0341	0.9640	0.0128	0.9414	0.0161	−9	−5	0.000
**White matter**	0.3743	0.0397	0.4207	0.0175	0.4246	0.1753	−11	−11	0.000
**Gray matter**	0.5214	0.0398	0.5433	0.0167	0.5168	0.1521	0	0	0.000
**Cortical gray matter**	0.3391	0.0257	0.3702	0.1404	0.3382	0.0509	−13	0	0.000
**Frontal lobe**									
Total	0.1271	0.0118	0.1333	0.0074	0.1305	0.0085	−6	−3	0.005
Right	0.0653	0.0060	0.0674	0.0039	0.0659	0.0039	0	0	0.028
Left	0.0622	0.0060	0.0659	0.0036	0.0646	0.0048	−7	0	0.001
**Parietal lobe**									
Total	0.0693	0.0095	0.0731	0.0037	0.0699	0.0041	−6	0	0.009
Right	0.0342	0.0050	0.0356	0.0019	0.0345	0.0026	−13	0	0.204
Left	0.0352	0.0048	0.0376	0.0020	0.0355	0.0021	0	0	0.000
**Precuneus lobe**									
Total	0.0151	0.0049	0.0178	0.0015	0.0162	0.0017	0	1	0.000
Right	0.0071	0.0026	0.0087	0.0008	0.0081	0.0010	−24	−16	0.009
Left	0.0070	0.0024	0.0091	0.0008	0.0081	0.0009	−29	−16	0.000
**Occipital lobe**									
Total	0.0502	0.0086	0.0536	0.0038	0.0519	0.0047	−7	−4	0.063
Right	0.0243	0.0042	0.0257	0.0019	0.0250	0.0025	−7	−3	0.243
Left	0.0263	0.0047	0.0279	0.0023	0.0269	0.0026	−6	−3	0.042
**Temporal lobe**									
Total	0.0944	0.0072	0.1102	0.0046	0.0929	0.0067	−17	1	0.000
Right	0.0484	0.0050	0.0561	0.0027	0.0469	0.0038	−17	8	0.000
Left	0.0470	0.0035	0.0540	0.0025	0.0459	0.0038	−15	3	0.000
**Hippocampus**									
Total	0.0050	0.0008	0.0057	0.0005	0.0053	0.0040	−17	−8	0.000
Right	0.0021	0.0004	0.0030	0.0003	0.0031	0.0039	−43	−49	0.000
Left	0.0022	0.0004	0.0027	0.0002	0.0022	0.0004	−23	0	0.000
**Parahippocampus**									
Total	0.0041	0.0011	0.0055	0.0005	0.0049	0.0006	−34	−20	0.000
Right	0.0023	0.0006	0.0027	0.0003	0.0024	0.0003	−17	−4	0.000
Left	0.0024	0.0005	0.0028	0.0002	0.0025	0.0003	−17	−4	0.000
**Entorhinal lobe**									
Total	0.0031	0.0007	0.0040	0.0005	0.0031	0.0006	−29	0	0.000
Right	0.0022	0.0004	0.0020	0.0003	0.0015	0.0003	9	27	0.000
Left	0.0021	0.0003	0.0020	0.0003	0.0016	0.0003	5	24	0.000
**Caudate**									
Total	0.0051	0.0018	0.0051	0.0010	0.0053	0.0012	0	−4	0.748
Right	0.0032	0.0010	0.0027	0.0005	0.0028	0.0007	16	13	0.985
Left	0.0021	0.0009	0.0024	0.0005	0.0025	0.0006	−14	−19	0.478
**Putamen**									
Total	0.0061	0.0014	0.0067	0.0007	0.0068	0.0008	−9	−11	0.000
Right	0.0031	0.0007	0.0033	0.0004	0.0034	0.0004	−6	−10	0.001
Left	0.0032	0.0008	0.0034	0.0004	0.0034	0.0005	−6	−6	0.002
**Pallidum**									
Total	0.0022	0.0004	0.0022	0.0002	0.0023	0.0002	0	−4	0.000
Right	0.0012	0.0002	0.0011	0.0001	0.0012	0.0001	9	0	0.000
Left	0.0013	0.0002	0.0011	0.0001	0.0012	0.0001	15	8	0.002
**Thalamus**									
Total	0.0111	0.0023	0.0124	0.0008	0.0115	0.0094	−11	−3	0.000
Right	0.0053	0.0012	0.0060	0.0004	0.0063	0.0003	−13	−19	0.000
Left	0.0052	0.0012	0.0064	0.0004	0.0079	0.0097	−23	−52	0.000
**Brainstem**	0.0211	0.0025	0.0223	0.0016	0.0243	0.0019	−6	−15	0.000
**Mesencephalon**	0.0063	0.0008	0.0062	0.0004	0.0073	0.0031	2	−16	0.000
**Pons**	0.0114	0.0016	0.0127	0.0032	0.0129	0.0022	−12	−13	0.000
**Cerebellum**	0.0743	0.0083	0.0846	0.0068	0.0846	0.0156	−14	−14	0.000
**Ventricle volume**	0.1042	0.0341	0.0360	0.0128	0.0586	0.0161	65	44	0.000
**Lateral ventricle**									
Total	0.1001	0.0335	0.0335	0.0125	0.0556	0.0159	67	44	0.000
Right	0.0491	0.0159	0.0157	0.0063	0.0274	0.0089	68	44	0.000
Left	0.0523	0.0185	0.0178	0.0065	0.0283	0.0085	66	46	0.000
**Third ventricle**	0.0022	0.0006	0.0013	0.0004	0.0016	0.0004	41	27	0.000
**Fourth ventricle**	0.0022	0.0006	0.0012	0.003	0.0013	0.0003	45	41	0.003

Mean relative volumes of the different brain volumes for iNPH, control group and AD patients. All volumes have been normalized to the intracranial volume (ICV). ^#^ *p*-value of the Kruskal–Wallis test. *p*-value < 0.005 was considered statistically significant. % Δ percentage difference of the mean of iNPH patients to the mean of healthy patients in the control group (CG) and to the mean of AD patients. Abbreviations: SD = standard deviation. ICV = intracranial volume. iNPH = idiopathic normal pressure hydrocephalus. AD = Alzheimer’s disease. CG = control group.

**Table 5:** Subgroup comparisons of absolute volumetric measurements in iNPH and AD patients and the healthy control group (CG).

Variable	Subgroups iNPH, CG, and AD	Mean Difference	SE	*p*
total ventricle volume	iNPH vs. CG	86.60	7.16	0.000 *
	iNPH vs. AD	64.11	7.16	0.000 *
	CG vs. AD	22.49	7.16	0.006 *
total lateral ventricle volume	iNPH vs. CG	85.05	7.02	0.006 *
	iNPH vs. AD	62.85	7.02	0.000 *
	CG vs. AD	22.20	7.02	0.006 *
right ventricle volume	iNPH vs. CG	41.94	3.41	0.000 *
	iNPH vs. AD	30.10	3.41	0.000 *
	CG vs. AD	11.84	3.41	0.002 *
left ventricle volume	iNPH vs. CG	43.11	3.80	0.000 *
	iNPH vs. AD	32.76	3.80	0.000 *
	CG vs. AD	10.36	3.80	0.022 *
third ventricle volume	iNPH vs. CG	0.95	0.13	0.000 *
	iNPH vs. AD	0.69	0.13	0.000 *
	CG vs. AD	0.27	0.13	0.147
fourth ventricle volume	iNPH vs. CG	0.60	0.12	0.000 *
	iNPH vs. AD	0.58	0.12	0.000 *
	CG vs. AD	0.02	0.12	1.000
total brain volume	iNPH vs. CG	−35.95	24.15	0.418
	iNPH vs. AD	51.74	24.15	0.103
	CG vs. AD	−87.69	24.15	0.001 *
white matter	iNPH vs. CG	−34.80	14.04	0.044 *
	iNPH vs. AD	−12.17	14.04	1.000
	CG vs. AD	−22.62	14.04	0.329
cortical gray matter	iNPH vs. CG	−20.10	11.85	0.277
	iNPH vs. AD	39.21	11.85	0.004 *
	CG vs. AD	−59.31	11.85	0.000 *
frontal lobe right	iNPH vs. CG	−0.03	1.95	1.000
	iNPH vs. AD	6.04	1.95	0.007 *
	CG vs. AD	−6.07	1.95	0.007 *
frontal lobe left	iNPH vs. CG	−1.79	1.90	1.000
	iNPH vs. AD	3.93	1.90	0.120
	CG vs. AD	−5.73	1.90	0.009 *
parietal lobe right	iNPH vs. CG	−0.166	1.15	1.000
	iNPH vs. AD	3.35	1.15	0.013 *
	CG vs. AD	−3.51	1.15	0.008 *
parietal lobe left	iNPH vs. CG	−1.30	1.16	0.786
	iNPH vs. AD	3.42	1.16	0.011 *
	CG vs. AD	−4.72	1.16	0.000 *
precuneus lobe right	iNPH vs. CG	−1.39	0.46	0.009 *
	iNPH vs. AD	−0.20	0.46	1.000
	CG vs. AD	−1.19	0.46	0.033 *
precuneus lobe left	iNPH vs. CG	−1.70	0.46	0.001 *
	iNPH vs. AD	−0.027	0.46	1.000
	CG vs. AD	−1.67	0.46	0.001 *
temporal lobe right	iNPH vs. CG	−7.50	1.69	0.000 *
	iNPH vs. AD	6.28	1.69	0.001 *
	CG vs. AD	−13.78	1.69	0.000 *
temporal lobe left	iNPH vs. CG	−6.58	1.51	0.000 *
	iNPH vs. AD	5.96	1.51	0.000 *
	CG vs. AD	−12.54	1.51	0.000 *

*, statistically significant at *p* < 0.05. Mean difference given in units of mL. SE, standard error. Abbreviations: iNPH = idiopathic normal pressure hydrocephalus. AD = Alzheimer’s disease. CG = control group.

**Table 6:** Subgroup comparisons of volumetric analysis in iNPH patients (iNPH) and AD patients and the control group in relative volumes, normalized to the total intracranial volume (ICV) of each subgroup.

Variable	Subgroups iNPH, CG, and AD	Mean	SE	*p*
total ventricle volume	iNPH vs. CG	129.35 vs. 42.75	50.03 vs. 16.51	0.000 *
	iNPH vs. AD	129.35 vs. 65.24	50.03 vs. 19.46	0.000 *
total lateral ventricle volume	iNPH vs. CG	124.85 vs. 39.80	49.02 vs. 16.03	0.000 *
	iNPH vs. AD	124.85 vs. 62.00	49.02 vs. 19.15	0.000 *
right ventricle volume	iNPH vs. CG	60.62 vs. 18.68	23.07 vs. 8.02	0.000 *
	iNPH vs. AD	60.62 vs. 30.52	23.07 vs. 10.83	0.000 *
left ventricle volume	iNPH vs. CG	64.23 vs. 21.12	26.90 vs. 8.31	0.000 *
	iNPH vs. AD	64.23 vs. 31.47	26.90 vs. 9.79	0.000 *
third ventricle volume	iNPH vs. CG	2.49 vs. 1.54	0.78 vs. 0.51	0.000 *
	iNPH vs. AD	2.49 vs. 1.80	0.78 vs. 0.48	0.000 *
fourth ventricle volume	iNPH vs. CG	2.01 vs. 1.41	0.81 vs. 0.40	0.000 *
	iNPH vs. AD	2.01 vs. 1.44	0.81 vs. 0.34	0.000 *
total brain volume	iNPH vs. CG	1102.46 vs. 1138.41	122.28 vs. 93.27	0.206
	iNPH vs. AD	1102.46 vs. 1050.72	122.28 vs. 110.58	0.046 *
white matter	iNPH vs. CG	462.07 vs. 496.87	82.69 vs. 45.79	0.017 *
	iNPH vs. AD	462.07 vs. 474.24	82.69 vs. 56.44	0.319
gray matter	iNPH vs. CG	638.69 vs. 641.54	64.44 vs. 54.76	0.952
	iNPH vs. AD	638.69 vs. 576.48	64.44 vs. 58.69	0.000 *
cortical gray matter	iNPH vs. CG	417.17 vs. 437.27	46.51 vs. 39.62	0.070 *
	iNPH vs. AD	417.17 vs. 377.95	46.51 vs. 70.04	0.003 *
frontal lobe right	iNPH vs. CG	79.59 vs. 79.61	9.53 vs. 8.18	0.792
	iNPH vs. AD	79.59 vs. 73.55	9.53 vs. 8.72	0.003 *
frontal lobe left	iNPH vs. CG	75.99 vs. 77.78	9.28 vs. 7.59	0.603
	iNPH vs. AD	75.99 vs. 72.05	9.28 vs. 8.80	0.067
total frontal lobe	iNPH vs. CG	155.57 vs. 157.40	18.59 vs. 15.67	0.853
	iNPH vs. AD	155.57 vs. 145.60	18.59 vs. 17.31	0.012 *
parietal lobe right	iNPH vs. CG	41.82 vs. 41.98	6.47 vs. 3.99	0.842
	iNPH vs. AD	41.82 vs. 38.47	6.47 vs. 4.84	0.005 *
parietal lobe left	iNPH vs. CG	43.06 vs. 44.36	6.37 vs. 4.18	0.412
	iNPH vs. AD	43.06 vs. 39.64	6.37 vs. 4.94	0.004 *
total parietal lobe	iNPH vs. CG	84.88 vs. 86.35	12.39 vs. 8.03	0.767
	iNPH vs. AD	84.88 vs. 78.11	12.39 vs. 9.41	0.003 *
precuneus lobe right	iNPH vs. CG	8.89 vs. 10.29	3.05 vs. 1.22	0.073 *
	iNPH vs. AD	8.89 vs. 9.10	3.05 vs. 1.51	0.200
precuneus lobe left	iNPH vs. CG	9.00 vs. 10.70	2.91 vs. 1.34	0.003 *
	iNPH vs. AD	9.00 vs. 9.03	2.91 vs. 1.70	0.177
total precuneus lobe	iNPH vs. CG	17.90 vs. 20.99	5.88 vs. 2.49	0.013 *
	iNPH vs. AD	17.90 vs. 18.13	5.88 vs. 3.06	0.196
occipital lobe right	iNPH vs. CG	29.44 vs. 30.32	5.81 vs. 3.38	0.693
	iNPH vs. AD	29.44 vs. 27.94	5.81 vs. 4.20	0.053 *
occipital lobe left	iNPH vs. CG	31.23 vs. 33.04	6.15 vs. 4.07	0.235
	iNPH vs. AD	31.23 vs. 29.97	6.15 vs. 4.07	0.072
total occipital lobe	iNPH vs. CG	60.68 vs. 63.36	11.56 vs. 7.16	0.386
	iNPH vs. AD	60.68 vs. 57.91	11.56 vs. 8.01	0.068
temporal lobe right	iNPH vs. CG	58.80 vs. 66.30	9.03 vs. 6.29	0.000 *
	iNPH vs. AD	58.80 vs. 52.52	9.03 vs. 7.43	0.001 *
temporal lobe left	iNPH vs. CG	57.23 vs. 63.81	7.49 vs. 5.78	0.000 *
	iNPH vs. AD	57.23 vs. 51.27	7.49 vs. 7.11	0.001 *
total temporal lobe	iNPH vs. CG	116.03 vs. 130.11	15.60 vs. 11.73	0.000 *
	iNPH vs. AD	116.03 vs. 103.79	15.60 vs. 14.01	0.000 *
hippocampus right	iNPH vs. CG	2.99 vs. 3.57	0.57 vs. 0.42	0.000 *
	iNPH vs. AD	2.99 vs. 3.32	0.57 vs. 3.82	0.030 *
hippocampus lobe left	iNPH vs. CG	2.78 vs. 3.15	0.57 vs. 0.36	0.005 *
	iNPH vs. AD	2.78 vs. 2.48	0.57 vs. 0.46	0.005 *
total volume hippocampus	iNPH vs. CG	5.77 vs. 6.72	1.10 vs. 0.75	0.000 *
	iNPH vs. AD	5.77 vs. 5.79	1.10 vs. 3.83	0.015 *
gyrus parahippocampalis right	iNPH vs. CG	2.61 vs. 3.19	0.76 vs. 0.44	0.000 *
	iNPH vs. AD	2.61 vs. 2.64	0.76 vs. 0.44	0.372
gyrus parahippocampalis left	iNPH vs. CG	2.75 vs. 3.29	0.70 vs. 0.40	0.000 *
	iNPH vs. AD	2.75 vs. 2.78	0.70 vs. 0.39	0.424
total volume parahippocampal	iNPH vs. CG	5.37 vs. 6.48	1.41 vs. 0.80	0.000 *
	iNPH vs. AD	5.37 vs. 5.42	1.41 vs. 0.80	0.380
regio entorhinalis right	iNPH vs. CG	1.99 vs. 2.42	0.51 vs. 0.39	0.000 *
	iNPH vs. AD	1.99 vs. 1.73	0.51 vs. 0.41	0.008 *
regio entorhinalis left	iNPH vs. CG	1.99 vs. 2.34	0.45 vs. 0.36	0.001 *
	iNPH vs. AD	1.99 vs. 1.74	0.45 vs. 0.43	0.005 *
total volume entorhinal	iNPH vs. CG	3.97 vs. 4.76	0.93 vs. 0.70	0.000 *
	iNPH vs. AD	3.97 vs. 3.47	0.93 vs. 0.79	0.004 *
nucleus caudatus right	iNPH vs. CG	3.19 vs. 3.20	1.19 vs. 0.64	0.662
	iNPH vs. AD	3.19 vs. 3.08	1.19 vs. 0.81	0.330
nucleus caudatus left	iNPH vs. CG	2.90 vs. 2.87	1.06 vs. 0.63	0.411
	iNPH vs. AD	2.90 vs. 2.82	1.06 vs. 0.76	0.568
total volume nucleus caudatus	iNPH vs. CG	6.09 vs. 6.06	2.18 vs. 1.24	0.516
	iNPH vs. AD	6.09 vs. 5.90	2.18 vs. 1.51	0.441
putamen right	iNPH vs. CG	3.57 vs. 3.90	0.73 vs. 0.51	0.055 *
	iNPH vs. AD	3.57 vs. 3.74	0.73 vs. 0.57	0.521
putamen left	iNPH vs. CG	3.51 vs. 3.99	0.94 vs. 0.52	0.024 *
	iNPH vs. AD	3.51 vs. 3.79	0.94 vs. 0.63	0.318
total volume putamen	iNPH vs. CG	2.49 vs. 2.55	0.44 vs. 0.35	0.929
	iNPH vs. AD	2.49 vs. 2.60	0.44 vs. 0.35	0.496
pallidum right	iNPH vs. CG	1.21 vs. 1.26	0.23 vs. 0.20	0.756
	iNPH vs. AD	1.21 vs. 1.30	0.23 vs. 0.18	0.218
pallidum left	iNPH vs. CG	1.28 vs. 1.29	0.23 vs. 0.16	0.536
	iNPH vs. AD	1.28 vs. 1.30	0.23 vs. 0.18	0.996
total volume pallidum	iNPH vs. CG	2.49 vs. 2.55	0.44 vs. 0.35	0.929
	iNPH vs. AD	2.49 vs. 2.60	0.44 vs. 0.35	0.496
thalamus right	iNPH vs. CG	6.34 vs. 7.12	1.37 vs. 0.60	0.000 *
	iNPH vs. AD	6.34 vs. 7.00	1.37 vs. 0.68	0.007 *
thalamus left	iNPH vs. CG	6.40 vs. 7.49	1.36 vs. 0.61	0.000 *
	iNPH vs. AD	6.40 vs. 8.60	1.36 vs. 9.08	0.003 *
total volume thalamus	iNPH vs. CG	12.74 vs. 14.61	2.64 vs. 1.18	0.000 *
	iNPH vs. AD	12.74 vs. 15.60	2.64 vs. 9.03	0.002 *
brainstem volume	iNPH vs. CG	25.41 vs. 26.24	3.62 vs. 2.33	0.292
	iNPH vs. AD	25.41 vs. 27.05	3.62 vs. 2.69	0.026 *
mesencephalon volume	iNPH vs. CG	7.11 vs. 7.34	0.94 vs. 0.60	0.336
	iNPH vs. AD	7.11 vs. 8.17	0.94 vs. 3.50	0.009 *
pons volume	iNPH vs. CG	13.98 vs. 14.93	2.40 vs. 3.95	0.260
	iNPH vs. AD	13.98 vs. 14.30	2.40 vs. 2.57	0.165
cerebellum volume	iNPH vs. CG	90.56 vs. 99.76	11.14 vs. 9.84	0.000 *
	iNPH vs. AD	90.56 vs. 94.04	11.14 vs. 17.97	0.032 *

*, statistically significant at *p* < 0.05. Mean difference given in units of mL. SE, standard error.

**Table 7:** Diagnostic performance of Evans’ index, corpus callosum angle, and total ventricle volume in mL for diagnosing idiopathic normal pressure hydrocephalus.

Parameter	AUC	SE	*p*	CI1	CI2	Cut-Off	Sen	Spec	Acc
**Evans’ index ^a^**	0.935	0.023	<0.001	0.890	0.979	0.3	0.829	0.951	0.9
**Corpus callosum angle ^a^**	0.613	0.049	0.049	0.517	0.710	90	1	0.464	0.68
**Total ventricle volume ^a^**	0.939	0.012	<0.001	0.888	0.990	76.6	0.927	0.854	0.87

The optimal cut-off point of each ROC analysis was selected according to the maximum Youden index. Cut-off points are given unitless for Evans’ index, in units of degrees (°) for corpus callosum angle, and in units of mL for total ventricle volume. AUC—area under the curve, SE—standard error, *p*—significance level, CI—95% confidence interval, Sen—sensitivity (true positive rate), Spec—specificity (true negative rate), Acc—accuracy (rate of correctly identified cases). ^a^ Means that a higher test result indicates a more positive test.

## Data Availability

All relevant scientific data are included in the manuscript or the Supplementary Materials.

## Supplementary Materials

The following supporting information can be downloaded at https://www.mdpi.com/article/10.3390/diagnostics14131422/s1, Table S1: Subgroup comparisons in absolute volumes.
