# Supplementary material for: Brain and Ventricle Volume Alterations in Idiopathic Normal Pressure Hydrocephalus Determined by Artificial Intelligence-Based MRI Volumetry
Source: Diagnostics (Basel). 2024 Jul 3;14(13):1422. doi: 10.3390/diagnostics14131422 (PMC11241572; doi:10.3390/diagnostics14131422)
Supplement: Supplementary file 1 [file diagnostics-14-01422-s001.zip › diagnostics-3055683-supplementary.pdf]

## Subgroup comparisons in absolute volumes

| dependent variable | (I) NPH | (J) NPH | mean difference (I-J) | Std.-error | Sig. | 95% confidence interval Minimum | Maximum |
|---|---|---|---|---|---|---|---|
| total_ventricle_volume | 0=no iNPH | 1=iNPH | -86,60000* | 7,16190 | ,000 | -103,9891 | -69,2109 |
| | | 2=AD | -22,48780* | 7,16190 | ,006 | -39,8769 | -5,0987 |
| | 1=iNPH | 0=no iNPH | 86,60000* | 7,16190 | ,000 | 69,2109 | 103,9891 |
| | | 2=AD | 64,11220* | 7,16190 | ,000 | 46,7231 | 81,5013 |
| | 2=AD | 0=no iNPH | 22,48780* | 7,16190 | ,006 | 5,0987 | 39,8769 |
| | | 1=iNPH | -64,11220* | 7,16190 | ,000 | -81,5013 | -46,7231 |
| total_lateral_ventricle_volume | 0=no iNPH | 1=iNPH | -85,04878* | 7,01611 | ,000 | -102,0839 | -68,0136 |
| | | 2=AD | -22,19756* | 7,01611 | ,006 | -39,2327 | -5,1624 |
| | 1=iNPH | 0=no iNPH | 85,04878* | 7,01611 | ,000 | 68,0136 | 102,0839 |
| | | 2=AD | 62,85122* | 7,01611 | ,000 | 45,8161 | 79,8864 |
| | 2=AD | 0=no iNPH | 22,19756* | 7,01611 | ,006 | 5,1624 | 39,2327 |
| | | 1=iNPH | -62,85122* | 7,01611 | ,000 | -79,8864 | -45,8161 |
| Left_ventricle_volume | 0=no iNPH | 1=iNPH | -43,11220* | 3,80059 | ,000 | -52,3400 | -33,8844 |
| | | 2=AD | -10,35610* | 3,80059 | ,022 | -19,5839 | -1,1283 |
| | 1=iNPH | 0=no iNPH | 43,11220* | 3,80059 | ,000 | 33,8844 | 52,3400 |
| | | 2=AD | 32,75610* | 3,80059 | ,000 | 23,5283 | 41,9839 |
| | 2=AD | 0=no iNPH | 10,35610* | 3,80059 | ,022 | 1,1283 | 19,5839 |
| | | 1=iNPH | -32,75610* | 3,80059 | ,000 | -41,9839 | -23,5283 |
| Right_ventricle_volume | 0=no iNPH | 1=iNPH | -41,93659* | 3,40716 | ,000 | -50,2092 | -33,6640 |
| | | 2=AD | -11,84146* | 3,40716 | ,002 | -20,1141 | -3,5689 |
| | 1=iNPH | 0=no iNPH | 41,93659* | 3,40716 | ,000 | 33,6640 | 50,2092 |
| | | 2=AD | 30,09512* | 3,40716 | ,000 | 21,8225 | 38,3677 |
| | 2=AD | 0=no iNPH | 11,84146* | 3,40716 | ,002 | 3,5689 | 20,1141 |
| | | 1=iNPH | -30,09512* | 3,40716 | ,000 | -38,3677 | -21,8225 |
| Third_ventricle_volume | 0=no iNPH | 1=iNPH | -,95122* | ,13370 | ,000 | -1,2758 | -,6266 |
| | | 2=AD | -,26585 | ,13370 | ,147 | -,5905 | ,0588 |
| | 1=iNPH | 0=no iNPH | ,95122* | ,13370 | ,000 | ,6266 | 1,2758 |
| | | 2=AD | ,68537* | ,13370 | ,000 | ,3607 | 1,0100 |
| | 2=AD | 0=no iNPH | ,26585 | ,13370 | ,147 | -,0588 | ,5905 |
| | | 1=iNPH | -,68537* | ,13370 | ,000 | -1,0100 | -,3607 |
| Fourth_ventricle_volume | 0=no iNPH | 1=iNPH | -,60000* | ,12238 | ,000 | -,8971 | -,3029 |
| | | 2=AD | -,02439 | ,12238 | 1,000 | -,3215 | ,2727 |
| | 1=iNPH | 0=no iNPH | ,60000* | ,12238 | ,000 | ,3029 | ,8971 |

| | | | | | | | |
|---|---|---|---|---|---|---|---|
| | | 2=AD | ,57561* | ,12238 | ,000 | ,2785 | ,8727 |
| | 2=AD | 0=no iNPH | ,02439 | ,12238 | 1,000 | -,2727 | ,3215 |
| | | 1=iNPH | -,57561* | ,12238 | ,000 | -,8727 | -,2785 |
| total_brain_volume | 0=no iNPH | 1=iNPH | 35,95366 | 24,15342 | ,418 | -22,6909 | 94,5983 |
| | | 2=AD | 87,69268* | 24,15342 | ,001 | 29,0481 | 146,3373 |
| | 1=iNPH | 0=no iNPH | -35,95366 | 24,15342 | ,418 | -94,5983 | 22,6909 |
| | | 2=AD | 51,73902 | 24,15342 | ,103 | -6,9056 | 110,3836 |
| | 2=AD | 0=no iNPH | -87,69268* | 24,15342 | ,001 | -146,3373 | -29,0481 |
| | | 1=iNPH | -51,73902 | 24,15342 | ,103 | -110,3836 | 6,9056 |
| White_matter | 0=no iNPH | 1=iNPH | 34,79756* | 14,03801 | ,044 | ,7132 | 68,8819 |
| | | 2=AD | 22,62439 | 14,03801 | ,329 | -11,4599 | 56,7087 |
| | 1=iNPH | 0=no iNPH | -34,79756* | 14,03801 | ,044 | -68,8819 | -,7132 |
| | | 2=AD | -12,17317 | 14,03801 | 1,000 | -46,2575 | 21,9112 |
| | 2=AD | 0=no iNPH | -22,62439 | 14,03801 | ,329 | -56,7087 | 11,4599 |
| | | 1=iNPH | 12,17317 | 14,03801 | 1,000 | -21,9112 | 46,2575 |
| Gray_matter | 0=no iNPH | 1=iNPH | 2,84878 | 13,12608 | 1,000 | -29,0214 | 34,7190 |
| | | 2=AD | 65,06098* | 13,12608 | ,000 | 33,1908 | 96,9312 |
| | 1=iNPH | 0=no iNPH | -2,84878 | 13,12608 | 1,000 | -34,7190 | 29,0214 |
| | | 2=AD | 62,21220* | 13,12608 | ,000 | 30,3420 | 94,0824 |
| | 2=AD | 0=no iNPH | -65,06098* | 13,12608 | ,000 | -96,9312 | -33,1908 |
| | | 1=iNPH | -62,21220* | 13,12608 | ,000 | -94,0824 | -30,3420 |
| Cortical_gray_matter | 0=no iNPH | 1=iNPH | 20,10000 | 11,85179 | ,277 | -8,6762 | 48,8762 |
| | | 2=AD | 59,31463* | 11,85179 | ,000 | 30,5384 | 88,0908 |
| | 1=iNPH | 0=no iNPH | -20,10000 | 11,85179 | ,277 | -48,8762 | 8,6762 |
| | | 2=AD | 39,21463* | 11,85179 | ,004 | 10,4384 | 67,9908 |
| | 2=AD | 0=no iNPH | -59,31463* | 11,85179 | ,000 | -88,0908 | -30,5384 |
| | | 1=iNPH | -39,21463* | 11,85179 | ,004 | -67,9908 | -10,4384 |
| total_volume_frontal_lobe | 0=no iNPH | 1=iNPH | 1,82195 | 3,80586 | 1,000 | -7,4187 | 11,0626 |
| | | 2=AD | 11,79512* | 3,80586 | ,007 | 2,5545 | 21,0358 |
| | 1=iNPH | 0=no iNPH | -1,82195 | 3,80586 | 1,000 | -11,0626 | 7,4187 |
| | | 2=AD | 9,97317* | 3,80586 | ,030 | ,7325 | 19,2138 |
| | 2=AD | 0=no iNPH | -11,79512* | 3,80586 | ,007 | -21,0358 | -2,5545 |
| | | 1=iNPH | -9,97317* | 3,80586 | ,030 | -19,2138 | -,7325 |
| Frontal_right_volume | 0=no iNPH | 1=iNPH | ,02927 | 1,94938 | 1,000 | -4,7038 | 4,7624 |
| | | 2=AD | 6,06829* | 1,94938 | ,007 | 1,3352 | 10,8014 |
| | 1=iNPH | 0=no iNPH | -,02927 | 1,94938 | 1,000 | -4,7624 | 4,7038 |
| | | 2=AD | 6,03902* | 1,94938 | ,007 | 1,3059 | 10,7721 |
| | 2=AD | 0=no iNPH | -6,06829* | 1,94938 | ,007 | -10,8014 | -1,3352 |

| | | | | | | | |
|---|---|---|---|---|---|---|---|
| | | 1=iNPH | -6,03902* | 1,94938 | ,007 | -10,7721 | -1,3059 |
| Frontal_left_volume | 0=no iNPH | 1=iNPH | 1,79268 | 1,89631 | 1,000 | -2,8116 | 6,3969 |
| | | 2=AD | 5,72683* | 1,89631 | ,009 | 1,1226 | 10,3311 |
| | 1=iNPH | 0=no iNPH | -1,79268 | 1,89631 | 1,000 | -6,3969 | 2,8116 |
| | | 2=AD | 3,93415 | 1,89631 | ,120 | -,6701 | 8,5384 |
| | 2=AD | 0=no iNPH | -5,72683* | 1,89631 | ,009 | -10,3311 | -1,1226 |
| | | 1=iNPH | -3,93415 | 1,89631 | ,120 | -8,5384 | ,6701 |
| total_volume_parietal_lobe | 0=no iNPH | 1=iNPH | 1,47073 | 2,23226 | 1,000 | -3,9492 | 6,8907 |
| | | 2=AD | 8,23659* | 2,23226 | ,001 | 2,8166 | 13,6565 |
| | 1=iNPH | 0=no iNPH | -1,47073 | 2,23226 | 1,000 | -6,8907 | 3,9492 |
| | | 2=AD | 6,76585* | 2,23226 | ,009 | 1,3459 | 12,1858 |
| | 2=AD | 0=no iNPH | -8,23659* | 2,23226 | ,001 | -13,6565 | -2,8166 |
| | | 1=iNPH | -6,76585* | 2,23226 | ,009 | -12,1858 | -1,3459 |
| Parietal_right_volume | 0=no iNPH | 1=iNPH | ,16585 | 1,14935 | 1,000 | -2,6248 | 2,9565 |
| | | 2=AD | 3,51220* | 1,14935 | ,008 | ,7216 | 6,3028 |
| | 1=iNPH | 0=no iNPH | -,16585 | 1,14935 | 1,000 | -2,9565 | 2,6248 |
| | | 2=AD | 3,34634* | 1,14935 | ,013 | ,5557 | 6,1370 |
| | 2=AD | 0=no iNPH | -3,51220* | 1,14935 | ,008 | -6,3028 | -,7216 |
| | | 1=iNPH | -3,34634* | 1,14935 | ,013 | -6,1370 | -,5557 |
| Parietal_left_volume | 0=no iNPH | 1=iNPH | 1,30488 | 1,15811 | ,786 | -1,5070 | 4,1168 |
| | | 2=AD | 4,72439* | 1,15811 | ,000 | 1,9125 | 7,5363 |
| | 1=iNPH | 0=no iNPH | -1,30488 | 1,15811 | ,786 | -4,1168 | 1,5070 |
| | | 2=AD | 3,41951* | 1,15811 | ,011 | ,6076 | 6,2314 |
| | 2=AD | 0=no iNPH | -4,72439* | 1,15811 | ,000 | -7,5363 | -1,9125 |
| | | 1=iNPH | -3,41951* | 1,15811 | ,011 | -6,2314 | -,6076 |
| total_volume_precuneus | 0=no iNPH | 1=iNPH | 3,09268* | ,90257 | ,003 | ,9012 | 5,2841 |
| | | 2=AD | 2,86341* | ,90257 | ,006 | ,6720 | 5,0549 |
| | 1=iNPH | 0=no iNPH | -3,09268* | ,90257 | ,003 | -5,2841 | -,9012 |
| | | 2=AD | -,22927 | ,90257 | 1,000 | -2,4207 | 1,9622 |
| | 2=AD | 0=no iNPH | -2,86341* | ,90257 | ,006 | -5,0549 | -,6720 |
| | | 1=iNPH | ,22927 | ,90257 | 1,000 | -1,9622 | 2,4207 |
| Precuneus_right_volume | 0=no iNPH | 1=iNPH | 1,39268* | ,46123 | ,009 | ,2728 | 2,5125 |
| | | 2=AD | 1,19024* | ,46123 | ,033 | ,0704 | 2,3101 |
| | 1=iNPH | 0=no iNPH | -1,39268* | ,46123 | ,009 | -2,5125 | -,2728 |
| | | 2=AD | -,20244 | ,46123 | 1,000 | -1,3223 | ,9174 |
| | 2=AD | 0=no iNPH | -1,19024* | ,46123 | ,033 | -2,3101 | -,0704 |
| | | 1=iNPH | ,20244 | ,46123 | 1,000 | -,9174 | 1,3223 |
| Precuneus_left_volume | 0=no iNPH | 1=iNPH | 1,70000* | ,46170 | ,001 | ,5790 | 2,8210 |

| | | | | | | | |
|---|---|---|---|---|---|---|---|
| | | 2=AD | 1,67317* | ,46170 | ,001 | ,5522 | 2,7942 |
| | 1=iNPH | 0=no iNPH | -1,70000* | ,46170 | ,001 | -2,8210 | -,5790 |
| | | 2=AD | -,02683 | ,46170 | 1,000 | -1,1478 | 1,0942 |
| | 2=AD | 0=no iNPH | -1,67317* | ,46170 | ,001 | -2,7942 | -,5522 |
| | | 1=iNPH | ,02683 | ,46170 | 1,000 | -1,0942 | 1,1478 |
| total_volume_occipital | 0=no iNPH | 1=iNPH | 2,68537 | 2,01236 | ,554 | -2,2006 | 7,5714 |
| | | 2=AD | 5,44634* | 2,01236 | ,023 | ,5603 | 10,3324 |
| | 1=iNPH | 0=no iNPH | -2,68537 | 2,01236 | ,554 | -7,5714 | 2,2006 |
| | | 2=AD | 2,76098 | 2,01236 | ,518 | -2,1250 | 7,6470 |
| | 2=AD | 0=no iNPH | -5,44634* | 2,01236 | ,023 | -10,3324 | -,5603 |
| | | 1=iNPH | -2,76098 | 2,01236 | ,518 | -7,6470 | 2,1250 |
| Occipital_right_volume | 0=no iNPH | 1=iNPH | ,87805 | 1,01004 | 1,000 | -1,5743 | 3,3304 |
| | | 2=AD | 2,37805 | 1,01004 | ,061 | -,0743 | 4,8304 |
| | 1=iNPH | 0=no iNPH | -,87805 | 1,01004 | 1,000 | -3,3304 | 1,5743 |
| | | 2=AD | 1,50000 | 1,01004 | ,420 | -,9524 | 3,9524 |
| | 2=AD | 0=no iNPH | -2,37805 | 1,01004 | ,061 | -4,8304 | ,0743 |
| | | 1=iNPH | -1,50000 | 1,01004 | ,420 | -3,9524 | ,9524 |
| Occipital_left_volume | 0=no iNPH | 1=iNPH | 1,80732 | 1,07402 | ,285 | -,8004 | 4,4150 |
| | | 2=AD | 3,06829* | 1,07402 | ,015 | ,4606 | 5,6760 |
| | 1=iNPH | 0=no iNPH | -1,80732 | 1,07402 | ,285 | -4,4150 | ,8004 |
| | | 2=AD | 1,26098 | 1,07402 | ,728 | -1,3468 | 3,8687 |
| | 2=AD | 0=no iNPH | -3,06829* | 1,07402 | ,015 | -5,6760 | -,4606 |
| | | 1=iNPH | -1,26098 | 1,07402 | ,728 | -3,8687 | 1,3468 |
| total_volume_temporal | 0=no iNPH | 1=iNPH | 14,08049* | 3,06303 | ,000 | 6,6434 | 21,5175 |
| | | 2=AD | 26,31707* | 3,06303 | ,000 | 18,8800 | 33,7541 |
| | 1=iNPH | 0=no iNPH | -14,08049* | 3,06303 | ,000 | -21,5175 | -6,6434 |
| | | 2=AD | 12,23659* | 3,06303 | ,000 | 4,7995 | 19,6736 |
| | 2=AD | 0=no iNPH | -26,31707* | 3,06303 | ,000 | -33,7541 | -18,8800 |
| | | 1=iNPH | -12,23659* | 3,06303 | ,000 | -19,6736 | -4,7995 |
| Temporal_right_volume | 0=no iNPH | 1=iNPH | 7,49756* | 1,69318 | ,000 | 3,3865 | 11,6086 |
| | | 2=AD | 13,77805* | 1,69318 | ,000 | 9,6670 | 17,8891 |
| | 1=iNPH | 0=no iNPH | -7,49756* | 1,69318 | ,000 | -11,6086 | -3,3865 |
| | | 2=AD | 6,28049* | 1,69318 | ,001 | 2,1694 | 10,3915 |
| | 2=AD | 0=no iNPH | -13,77805* | 1,69318 | ,000 | -17,8891 | -9,6670 |
| | | 1=iNPH | -6,28049* | 1,69318 | ,001 | -10,3915 | -2,1694 |
| Temporal_left_volume | 0=no iNPH | 1=iNPH | 6,58293* | 1,50973 | ,000 | 2,9173 | 10,2486 |
| | | 2=AD | 12,53902* | 1,50973 | ,000 | 8,8734 | 16,2047 |
| | 1=iNPH | 0=no iNPH | -6,58293* | 1,50973 | ,000 | -10,2486 | -2,9173 |

| | | | | | | | |
|---|---|---|---|---|---|---|---|
| | | 2=AD | 5,95610* | 1,50973 | ,000 | 2,2905 | 9,6217 |
| | 2=AD | 0=no iNPH | -12,53902* | 1,50973 | ,000 | -16,2047 | -8,8734 |
| | | 1=iNPH | -5,95610* | 1,50973 | ,000 | -9,6217 | -2,2905 |
| total_volume_Hippocampus | 0=no iNPH | 1=iNPH | ,94878 | ,51664 | ,206 | -,3056 | 2,2032 |
| | | 2=AD | ,92439 | ,51664 | ,228 | -,3300 | 2,1788 |
| | 1=iNPH | 0=no iNPH | -,94878 | ,51664 | ,206 | -2,2032 | ,3056 |
| | | 2=AD | -,02439 | ,51664 | 1,000 | -1,2788 | 1,2300 |
| | 2=AD | 0=no iNPH | -,92439 | ,51664 | ,228 | -2,1788 | ,3300 |
| | | 1=iNPH | ,02439 | ,51664 | 1,000 | -1,2300 | 1,2788 |
| Hippocampus_right_volume | 0=no iNPH | 1=iNPH | ,58293 | ,49529 | ,725 | -,6196 | 1,7855 |
| | | 2=AD | ,25122 | ,49529 | 1,000 | -,9513 | 1,4538 |
| | 1=iNPH | 0=no iNPH | -,58293 | ,49529 | ,725 | -1,7855 | ,6196 |
| | | 2=AD | -,33171 | ,49529 | 1,000 | -1,5343 | ,8708 |
| | 2=AD | 0=no iNPH | -,25122 | ,49529 | 1,000 | -1,4538 | ,9513 |
| | | 1=iNPH | ,33171 | ,49529 | 1,000 | -,8708 | 1,5343 |
| Hippocampus_left_volume | 0=no iNPH | 1=iNPH | ,36585* | ,10433 | ,002 | ,1125 | ,6192 |
| | | 2=AD | ,67317* | ,10433 | ,000 | ,4199 | ,9265 |
| | 1=iNPH | 0=no iNPH | -,36585* | ,10433 | ,002 | -,6192 | -,1125 |
| | | 2=AD | ,30732* | ,10433 | ,012 | ,0540 | ,5606 |
| | 2=AD | 0=no iNPH | -,67317* | ,10433 | ,000 | -,9265 | -,4199 |
| | | 1=iNPH | -,30732* | ,10433 | ,012 | -,5606 | -,0540 |
| total_volume_parahippocampal | 0=no iNPH | 1=iNPH | 1,10732* | ,23083 | ,000 | ,5469 | 1,6678 |
| | | 2=AD | 1,05366* | ,23083 | ,000 | ,4932 | 1,6141 |
| | 1=iNPH | 0=no iNPH | -1,10732* | ,23083 | ,000 | -1,6678 | -,5469 |
| | | 2=AD | -,05366 | ,23083 | 1,000 | -,6141 | ,5068 |
| | 2=AD | 0=no iNPH | -1,05366* | ,23083 | ,000 | -1,6141 | -,4932 |
| | | 1=iNPH | ,05366 | ,23083 | 1,000 | -,5068 | ,6141 |
| Gyrus_parahippocampalis_right_volume | 0=no iNPH | 1=iNPH | ,57561* | ,12472 | ,000 | ,2728 | ,8784 |
| | | 2=AD | ,54634* | ,12472 | ,000 | ,2435 | ,8492 |
| | 1=iNPH | 0=no iNPH | -,57561* | ,12472 | ,000 | -,8784 | -,2728 |
| | | 2=AD | -,02927 | ,12472 | 1,000 | -,3321 | ,2736 |
| | 2=AD | 0=no iNPH | -,54634* | ,12472 | ,000 | -,8492 | -,2435 |
| | | 1=iNPH | ,02927 | ,12472 | 1,000 | -,2736 | ,3321 |
| Gyrus_parahippocampalis_left_volume | 0=no iNPH | 1=iNPH | ,53171* | ,11481 | ,000 | ,2530 | ,8105 |
| | | 2=AD | ,50732* | ,11481 | ,000 | ,2286 | ,7861 |
| | 1=iNPH | 0=no iNPH | -,53171* | ,11481 | ,000 | -,8105 | -,2530 |
| | | 2=AD | -,02439 | ,11481 | 1,000 | -,3031 | ,2544 |
| | 2=AD | 0=no iNPH | -,50732* | ,11481 | ,000 | -,7861 | -,2286 |

| | | | | | | | |
|---|---|---|---|---|---|---|---|
| | | 1=iNPH | ,02439 | ,11481 | 1,000 | -,2544 | ,3031 |
| total_volume_entorhinalis | 0=no iNPH | 1=iNPH | ,78537* | ,17961 | ,000 | ,3493 | 1,2215 |
| | | 2=AD | 1,28537* | ,17961 | ,000 | ,8493 | 1,7215 |
| | 1=iNPH | 0=no iNPH | -,78537* | ,17961 | ,000 | -1,2215 | -,3493 |
| | | 2=AD | ,50000* | ,17961 | ,019 | ,0639 | ,9361 |
| | 2=AD | 0=no iNPH | -1,28537* | ,17961 | ,000 | -1,7215 | -,8493 |
| | | 1=iNPH | -,50000* | ,17961 | ,019 | -,9361 | -,0639 |
| Regio_entorhinalis_right_volume | 0=no iNPH | 1=iNPH | ,43415* | ,09683 | ,000 | ,1990 | ,6693 |
| | | 2=AD | ,68780* | ,09683 | ,000 | ,4527 | ,9229 |
| | 1=iNPH | 0=no iNPH | -,43415* | ,09683 | ,000 | -,6693 | -,1990 |
| | | 2=AD | ,25366* | ,09683 | ,030 | ,0185 | ,4888 |
| | 2=AD | 0=no iNPH | -,68780* | ,09683 | ,000 | -,9229 | -,4527 |
| | | 1=iNPH | -,25366* | ,09683 | ,030 | -,4888 | -,0185 |
| Regio_entorhinalis_left_volume | 0=no iNPH | 1=iNPH | ,35122* | ,09116 | ,001 | ,1299 | ,5726 |
| | | 2=AD | ,59756* | ,09116 | ,000 | ,3762 | ,8189 |
| | 1=iNPH | 0=no iNPH | -,35122* | ,09116 | ,001 | -,5726 | -,1299 |
| | | 2=AD | ,24634* | ,09116 | ,024 | ,0250 | ,4677 |
| | 2=AD | 0=no iNPH | -,59756* | ,09116 | ,000 | -,8189 | -,3762 |
| | | 1=iNPH | -,24634* | ,09116 | ,024 | -,4677 | -,0250 |
| total_volume_Nucleu_caudatus | 0=no iNPH | 1=iNPH | -,02683 | ,37319 | 1,000 | -,9329 | ,8793 |
| | | 2=AD | ,15854 | ,37319 | 1,000 | -,7476 | 1,0646 |
| | 1=iNPH | 0=no iNPH | ,02683 | ,37319 | 1,000 | -,8793 | ,9329 |
| | | 2=AD | ,18537 | ,37319 | 1,000 | -,7207 | 1,0915 |
| | 2=AD | 0=no iNPH | -,15854 | ,37319 | 1,000 | -1,0646 | ,7476 |
| | | 1=iNPH | -,18537 | ,37319 | 1,000 | -1,0915 | ,7207 |
| Nucleus_caudatus_right_volume | 0=no iNPH | 1=iNPH | ,00976 | ,20038 | 1,000 | -,4768 | ,4963 |
| | | 2=AD | ,11707 | ,20038 | 1,000 | -,3695 | ,6036 |
| | 1=iNPH | 0=no iNPH | -,00976 | ,20038 | 1,000 | -,4963 | ,4768 |
| | | 2=AD | ,10732 | ,20038 | 1,000 | -,3792 | ,5938 |
| | 2=AD | 0=no iNPH | -,11707 | ,20038 | 1,000 | -,6036 | ,3695 |
| | | 1=iNPH | -,10732 | ,20038 | 1,000 | -,5938 | ,3792 |
| Nucleus_caudatus_left_volume | 0=no iNPH | 1=iNPH | -,03659 | ,18489 | 1,000 | -,4855 | ,4123 |
| | | 2=AD | ,04146 | ,18489 | 1,000 | -,4075 | ,4904 |
| | 1=iNPH | 0=no iNPH | ,03659 | ,18489 | 1,000 | -,4123 | ,4855 |
| | | 2=AD | ,07805 | ,18489 | 1,000 | -,3709 | ,5270 |
| | 2=AD | 0=no iNPH | -,04146 | ,18489 | 1,000 | -,4904 | ,4075 |
| | | 1=iNPH | -,07805 | ,18489 | 1,000 | -,5270 | ,3709 |
| total_volume_Putamen | 0=no iNPH | 1=iNPH | ,06098 | ,08417 | 1,000 | -,1434 | ,2653 |

| | | | | | | | |
|---|---|---|---|---|---|---|---|
| | | 2=AD | -,04634 | ,08417 | 1,000 | -,2507 | ,1580 |
| | 1=iNPH | 0=no iNPH | -,06098 | ,08417 | 1,000 | -,2653 | ,1434 |
| | | 2=AD | -,10732 | ,08417 | ,614 | -,3117 | ,0970 |
| | 2=AD | 0=no iNPH | ,04634 | ,08417 | 1,000 | -,1580 | ,2507 |
| | | 1=iNPH | ,10732 | ,08417 | ,614 | -,0970 | ,3117 |
| Putamen_right_volume | 0=no iNPH | 1=iNPH | ,33415* | ,13493 | ,044 | ,0065 | ,6617 |
| | | 2=AD | ,16098 | ,13493 | ,706 | -,1666 | ,4886 |
| | 1=iNPH | 0=no iNPH | -,33415* | ,13493 | ,044 | -,6617 | -,0065 |
| | | 2=AD | -,17317 | ,13493 | ,605 | -,5008 | ,1544 |
| | 2=AD | 0=no iNPH | -,16098 | ,13493 | ,706 | -,4886 | ,1666 |
| | | 1=iNPH | ,17317 | ,13493 | ,605 | -,1544 | ,5008 |
| Putamen_left_volume | 0=no iNPH | 1=iNPH | ,47317* | ,15816 | ,010 | ,0891 | ,8572 |
| | | 2=AD | ,20000 | ,15816 | ,625 | -,1840 | ,5840 |
| | 1=iNPH | 0=no iNPH | -,47317* | ,15816 | ,010 | -,8572 | -,0891 |
| | | 2=AD | -,27317 | ,15816 | ,260 | -,6572 | ,1109 |
| | 2=AD | 0=no iNPH | -,20000 | ,15816 | ,625 | -,5840 | ,1840 |
| | | 1=iNPH | ,27317 | ,15816 | ,260 | -,1109 | ,6572 |
| total_volume_Pallidum | 0=no iNPH | 1=iNPH | ,06098 | ,08417 | 1,000 | -,1434 | ,2653 |
| | | 2=AD | -,04634 | ,08417 | 1,000 | -,2507 | ,1580 |
| | 1=iNPH | 0=no iNPH | -,06098 | ,08417 | 1,000 | -,2653 | ,1434 |
| | | 2=AD | -,10732 | ,08417 | ,614 | -,3117 | ,0970 |
| | 2=AD | 0=no iNPH | ,04634 | ,08417 | 1,000 | -,1580 | ,2507 |
| | | 1=iNPH | ,10732 | ,08417 | ,614 | -,0970 | ,3117 |
| Pallidum_right_volume | 0=no iNPH | 1=iNPH | ,05366 | ,04477 | ,699 | -,0550 | ,1624 |
| | | 2=AD | -,03171 | ,04477 | 1,000 | -,1404 | ,0770 |
| | 1=iNPH | 0=no iNPH | -,05366 | ,04477 | ,699 | -,1624 | ,0550 |
| | | 2=AD | -,08537 | ,04477 | ,177 | -,1941 | ,0233 |
| | 2=AD | 0=no iNPH | ,03171 | ,04477 | 1,000 | -,0770 | ,1404 |
| | | 1=iNPH | ,08537 | ,04477 | ,177 | -,0233 | ,1941 |
| Pallidum_left_volume | 0=no iNPH | 1=iNPH | ,00732 | ,04228 | 1,000 | -,0953 | ,1100 |
| | | 2=AD | -,01463 | ,04228 | 1,000 | -,1173 | ,0880 |
| | 1=iNPH | 0=no iNPH | -,00732 | ,04228 | 1,000 | -,1100 | ,0953 |
| | | 2=AD | -,02195 | ,04228 | 1,000 | -,1246 | ,0807 |
| | 2=AD | 0=no iNPH | ,01463 | ,04228 | 1,000 | -,0880 | ,1173 |
| | | 1=iNPH | ,02195 | ,04228 | 1,000 | -,0807 | ,1246 |
| total_volume_Thalamus | 0=no iNPH | 1=iNPH | 1,86829 | 1,20843 | ,374 | -1,0658 | 4,8024 |
| | | 2=AD | -,98293 | 1,20843 | 1,000 | -3,9170 | 1,9512 |
| | 1=iNPH | 0=no iNPH | -1,86829 | 1,20843 | ,374 | -4,8024 | 1,0658 |

| | | | | | | | |
|---|---|---|---|---|---|---|---|
| | | 2=AD | -2,85122 | 1,20843 | ,060 | -5,7853 | ,0829 |
| | 2=AD | 0=no iNPH | ,98293 | 1,20843 | 1,000 | -1,9512 | 3,9170 |
| | | 1=iNPH | 2,85122 | 1,20843 | ,060 | -,0829 | 5,7853 |
| Thalamus_right_volume | 0=no iNPH | 1=iNPH | ,78537* | ,20931 | ,001 | ,2772 | 1,2936 |
| | | 2=AD | ,12927 | ,20931 | 1,000 | -,3789 | ,6375 |
| | 1=iNPH | 0=no iNPH | -,78537* | ,20931 | ,001 | -1,2936 | -,2772 |
| | | 2=AD | -,65610* | ,20931 | ,006 | -1,1643 | -,1479 |
| | 2=AD | 0=no iNPH | -,12927 | ,20931 | 1,000 | -,6375 | ,3789 |
| | | 1=iNPH | ,65610* | ,20931 | ,006 | ,1479 | 1,1643 |
| Thalamus_left_volume | 0=no iNPH | 1=iNPH | 1,08293 | 1,17300 | 1,000 | -1,7651 | 3,9310 |
| | | 2=AD | -1,11220 | 1,17300 | 1,000 | -3,9602 | 1,7359 |
| | 1=iNPH | 0=no iNPH | -1,08293 | 1,17300 | 1,000 | -3,9310 | 1,7651 |
| | | 2=AD | -2,19512 | 1,17300 | ,191 | -5,0432 | ,6529 |
| | 2=AD | 0=no iNPH | 1,11220 | 1,17300 | 1,000 | -1,7359 | 3,9602 |
| | | 1=iNPH | 2,19512 | 1,17300 | ,191 | -,6529 | 5,0432 |
| Brainstem_volume | 0=no iNPH | 1=iNPH | ,82195 | ,64729 | ,620 | -,7497 | 2,3936 |
| | | 2=AD | -,81707 | ,64729 | ,628 | -2,3887 | ,7546 |
| | 1=iNPH | 0=no iNPH | -,82195 | ,64729 | ,620 | -2,3936 | ,7497 |
| | | 2=AD | -1,63902* | ,64729 | ,038 | -3,2107 | -,0674 |
| | 2=AD | 0=no iNPH | ,81707 | ,64729 | ,628 | -,7546 | 2,3887 |
| | | 1=iNPH | 1,63902* | ,64729 | ,038 | ,0674 | 3,2107 |
| Mesencephalon_volume | 0=no iNPH | 1=iNPH | ,23171 | ,46847 | 1,000 | -,9057 | 1,3692 |
| | | 2=AD | -,83171 | ,46847 | ,235 | -1,9692 | ,3057 |
| | 1=iNPH | 0=no iNPH | -,23171 | ,46847 | 1,000 | -1,3692 | ,9057 |
| | | 2=AD | -1,06341 | ,46847 | ,075 | -2,2009 | ,0740 |
| | 2=AD | 0=no iNPH | ,83171 | ,46847 | ,235 | -,3057 | 1,9692 |
| | | 1=iNPH | 1,06341 | ,46847 | ,075 | -,0740 | 2,2009 |
| Pons_volume | 0=no iNPH | 1=iNPH | ,95122 | ,67468 | ,483 | -,6869 | 2,5893 |
| | | 2=AD | ,63659 | ,67468 | 1,000 | -1,0015 | 2,2747 |
| | 1=iNPH | 0=no iNPH | -,95122 | ,67468 | ,483 | -2,5893 | ,6869 |
| | | 2=AD | -,31463 | ,67468 | 1,000 | -1,9528 | 1,3235 |
| | 2=AD | 0=no iNPH | -,63659 | ,67468 | 1,000 | -2,2747 | 1,0015 |
| | | 1=iNPH | ,31463 | ,67468 | 1,000 | -1,3235 | 1,9528 |
| Cerebellar_grey_matter_volume | 0=no iNPH | 1=iNPH | 9,20000* | 2,97355 | ,007 | 1,9802 | 16,4198 |
| | | 2=AD | 5,72439 | 2,97355 | ,170 | -1,4954 | 12,9442 |
| | 1=iNPH | 0=no iNPH | -9,20000* | 2,97355 | ,007 | -16,4198 | -1,9802 |
| | | 2=AD | -3,47561 | 2,97355 | ,734 | -10,6954 | 3,7442 |
| | 2=AD | 0=no iNPH | -5,72439 | 2,97355 | ,170 | -12,9442 | 1,4954 |

| | | | | | | | |
|---|---|---|---|---|---|---|---|
| | | 1=iNPH | 3,47561 | 2,97355 | ,734 | -3,7442 | 10,6954 |

**Subgroup comparisons in relative volumes**

| dependent variable | (I) NPH | (J) NPH | Mean differences (I-J) | Std.- error | Sig. | 95% Confidence interval Minimum | Maximum |
|---|---|---|---|---|---|---|---|
| normalized_ventricle_volume | 0=no iNPH | 1=iNPH | -,06774* | ,00508 | ,000 | -,0801 | -,0554 |
| | | 2=AD | -,02254* | ,00508 | ,000 | -,0349 | -,0102 |
| | 1=iNPH | 0=no iNPH | ,06774* | ,00508 | ,000 | ,0554 | ,0801 |
| | | 2=AD | ,04521* | ,00508 | ,000 | ,0329 | ,0575 |
| | 2=AD | 0=no iNPH | ,02254* | ,00508 | ,000 | ,0102 | ,0349 |
| | | 1=iNPH | -,04521* | ,00508 | ,000 | -,0575 | -,0329 |
| normalized_lateral_ventricle | 0=no iNPH | 1=iNPH | -,06658* | ,00499 | ,000 | -,0787 | -,0545 |
| | | 2=AD | -,02212* | ,00499 | ,000 | -,0342 | -,0100 |
| | 1=iNPH | 0=no iNPH | ,06658* | ,00499 | ,000 | ,0545 | ,0787 |
| | | 2=AD | ,04445* | ,00499 | ,000 | ,0323 | ,0566 |
| | 2=AD | 0=no iNPH | ,02212* | ,00499 | ,000 | ,0100 | ,0342 |
| | | 1=iNPH | -,04445* | ,00499 | ,000 | -,0566 | -,0323 |
| normalized_right_lateral_ventricle | 0=no iNPH | 1=iNPH | -,03290* | ,00245 | ,000 | -,0389 | -,0269 |
| | | 2=AD | -,01163* | ,00245 | ,000 | -,0176 | -,0057 |
| | 1=iNPH | 0=no iNPH | ,03290* | ,00245 | ,000 | ,0269 | ,0389 |
| | | 2=AD | ,02127* | ,00245 | ,000 | ,0153 | ,0272 |
| | 2=AD | 0=no iNPH | ,01163* | ,00245 | ,000 | ,0057 | ,0176 |
| | | 1=iNPH | -,02127* | ,00245 | ,000 | -,0272 | -,0153 |
| normalized_left_lateral_ventricle | 0=no iNPH | 1=iNPH | -,03367* | ,00272 | ,000 | -,0403 | -,0271 |
| | | 2=AD | -,01049* | ,00272 | ,001 | -,0171 | -,0039 |
| | 1=iNPH | 0=no iNPH | ,03367* | ,00272 | ,000 | ,0271 | ,0403 |
| | | 2=AD | ,02318* | ,00272 | ,000 | ,0166 | ,0298 |
| | 2=AD | 0=no iNPH | ,01049* | ,00272 | ,001 | ,0039 | ,0171 |
| | | 1=iNPH | -,02318* | ,00272 | ,000 | -,0298 | -,0166 |
| normalized_third_ventricle | 0=no iNPH | 1=iNPH | -,00073* | ,00010 | ,000 | -,0010 | -,0005 |
| | | 2=AD | -,00032* | ,00010 | ,006 | -,0006 | -,0001 |
| | 1=iNPH | 0=no iNPH | ,00073* | ,00010 | ,000 | ,0005 | ,0010 |
| | | 2=AD | ,00040* | ,00010 | ,000 | ,0002 | ,0007 |
| | 2=AD | 0=no iNPH | ,00032* | ,00010 | ,006 | ,0001 | ,0006 |

| | | | | | | | |
|---|---|---|---|---|---|---|---|
| | | 1=iNPH | -,00040* | ,00010 | ,000 | -,0007 | -,0002 |
| normalized_fourth_ventricle | 0=no iNPH | 1=iNPH | -,02084* | ,00590 | ,002 | -,0352 | -,0065 |
| | | 2=AD | -,00481 | ,00590 | 1,000 | -,0191 | ,0095 |
| | 1=iNPH | 0=no iNPH | ,02084* | ,00590 | ,002 | ,0065 | ,0352 |
| | | 2=AD | ,01604* | ,00590 | ,023 | ,0017 | ,0304 |
| | 2=AD | 0=no iNPH | ,00481 | ,00590 | 1,000 | -,0095 | ,0191 |
| | | 1=iNPH | -,01604* | ,00590 | ,023 | -,0304 | -,0017 |
| normalized_total_brain_volume | 0=no iNPH | 1=iNPH | ,06774* | ,00508 | ,000 | ,0554 | ,0801 |
| | | 2=AD | ,02254* | ,00508 | ,000 | ,0102 | ,0349 |
| | 1=iNPH | 0=no iNPH | -,06774* | ,00508 | ,000 | -,0801 | -,0554 |
| | | 2=AD | -,04521* | ,00508 | ,000 | -,0575 | -,0329 |
| | 2=AD | 0=no iNPH | -,02254* | ,00508 | ,000 | -,0349 | -,0102 |
| | | 1=iNPH | ,04521* | ,00508 | ,000 | ,0329 | ,0575 |
| normalized_white_matter | 0=no iNPH | 1=iNPH | ,04676* | ,00596 | ,000 | ,0323 | ,0612 |
| | | 2=AD | -,00392 | ,00596 | 1,000 | -,0184 | ,0106 |
| | 1=iNPH | 0=no iNPH | -,04676* | ,00596 | ,000 | -,0612 | -,0323 |
| | | 2=AD | -,05068* | ,00596 | ,000 | -,0652 | -,0362 |
| | 2=AD | 0=no iNPH | ,00392 | ,00596 | 1,000 | -,0106 | ,0184 |
| | | 1=iNPH | ,05068* | ,00596 | ,000 | ,0362 | ,0652 |
| normalized_gray_matter | 0=no iNPH | 1=iNPH | ,02252* | ,00583 | ,001 | ,0084 | ,0367 |
| | | 2=AD | ,02645* | ,00583 | ,000 | ,0123 | ,0406 |
| | 1=iNPH | 0=no iNPH | -,02252* | ,00583 | ,001 | -,0367 | -,0084 |
| | | 2=AD | ,00393 | ,00583 | 1,000 | -,0102 | ,0181 |
| | 2=AD | 0=no iNPH | -,02645* | ,00583 | ,000 | -,0406 | -,0123 |
| | | 1=iNPH | -,00393 | ,00583 | 1,000 | -,0181 | ,0102 |
| normalized_cortical_gray_matter | 0=no iNPH | 1=iNPH | ,03035* | ,00749 | ,000 | ,0122 | ,0485 |
| | | 2=AD | ,03203* | ,00749 | ,000 | ,0138 | ,0502 |
| | 1=iNPH | 0=no iNPH | -,03035* | ,00749 | ,000 | -,0485 | -,0122 |
| | | 2=AD | ,00168 | ,00749 | 1,000 | -,0165 | ,0199 |
| | 2=AD | 0=no iNPH | -,03203* | ,00749 | ,000 | -,0502 | -,0138 |
| | | 1=iNPH | -,00168 | ,00749 | 1,000 | -,0199 | ,0165 |
| normalized_total_frontal_lobe | 0=no iNPH | 1=iNPH | ,00649* | ,00208 | ,007 | ,0014 | ,0115 |
| | | 2=AD | ,00276 | ,00208 | ,559 | -,0023 | ,0078 |
| | 1=iNPH | 0=no iNPH | -,00649* | ,00208 | ,007 | -,0115 | -,0014 |
| | | 2=AD | -,00373 | ,00208 | ,226 | -,0088 | ,0013 |
| | 2=AD | 0=no iNPH | -,00276 | ,00208 | ,559 | -,0078 | ,0023 |
| | | 1=iNPH | ,00373 | ,00208 | ,226 | -,0013 | ,0088 |
| normalized_frontal_right | 0=no iNPH | 1=iNPH | ,00256* | ,00104 | ,046 | ,0000 | ,0051 |

| | | | | | | | |
|---|---|---|---|---|---|---|---|
| | | 2=AD | ,00149 | ,00104 | ,465 | -,0010 | ,0040 |
| | 1=iNPH | 0=no iNPH | -,00256* | ,00104 | ,046 | -,0051 | ,0000 |
| | | 2=AD | -,00107 | ,00104 | ,920 | -,0036 | ,0015 |
| | 2=AD | 0=no iNPH | -,00149 | ,00104 | ,465 | -,0040 | ,0010 |
| | | 1=iNPH | ,00107 | ,00104 | ,920 | -,0015 | ,0036 |
| normalized_frontal_left | 0=no iNPH | 1=iNPH | ,00393* | ,00109 | ,001 | ,0013 | ,0066 |
| | | 2=AD | ,00127 | ,00109 | ,737 | -,0014 | ,0039 |
| | 1=iNPH | 0=no iNPH | -,00393* | ,00109 | ,001 | -,0066 | -,0013 |
| | | 2=AD | -,00266* | ,00109 | ,048 | -,0053 | ,0000 |
| | 2=AD | 0=no iNPH | -,00127 | ,00109 | ,737 | -,0039 | ,0014 |
| | | 1=iNPH | ,00266* | ,00109 | ,048 | ,0000 | ,0053 |
| normalized_total_parietal_lobe | 0=no iNPH | 1=iNPH | ,00378* | ,00139 | ,023 | ,0004 | ,0072 |
| | | 2=AD | ,00315 | ,00139 | ,077 | -,0002 | ,0065 |
| | 1=iNPH | 0=no iNPH | -,00378* | ,00139 | ,023 | -,0072 | -,0004 |
| | | 2=AD | -,00063 | ,00139 | 1,000 | -,0040 | ,0028 |
| | 2=AD | 0=no iNPH | -,00315 | ,00139 | ,077 | -,0065 | ,0002 |
| | | 1=iNPH | ,00063 | ,00139 | 1,000 | -,0028 | ,0040 |
| Normalize_parietal_right | 0=no iNPH | 1=iNPH | ,00137 | ,00076 | ,222 | -,0005 | ,0032 |
| | | 2=AD | ,00108 | ,00076 | ,479 | -,0008 | ,0029 |
| | 1=iNPH | 0=no iNPH | -,00137 | ,00076 | ,222 | -,0032 | ,0005 |
| | | 2=AD | -,00030 | ,00076 | 1,000 | -,0021 | ,0016 |
| | 2=AD | 0=no iNPH | -,00108 | ,00076 | ,479 | -,0029 | ,0008 |
| | | 1=iNPH | ,00030 | ,00076 | 1,000 | -,0016 | ,0021 |
| normalized_parietal_left | 0=no iNPH | 1=iNPH | ,00241* | ,00072 | ,003 | ,0007 | ,0041 |
| | | 2=AD | ,00208* | ,00072 | ,013 | ,0003 | ,0038 |
| | 1=iNPH | 0=no iNPH | -,00241* | ,00072 | ,003 | -,0041 | -,0007 |
| | | 2=AD | -,00033 | ,00072 | 1,000 | -,0021 | ,0014 |
| | 2=AD | 0=no iNPH | -,00208* | ,00072 | ,013 | -,0038 | -,0003 |
| | | 1=iNPH | ,00033 | ,00072 | 1,000 | -,0014 | ,0021 |
| normalized_total_precuneus_lobe | 0=no iNPH | 1=iNPH | ,00304* | ,00068 | ,000 | ,0014 | ,0047 |
| | | 2=AD | ,00157 | ,00068 | ,071 | -,0001 | ,0032 |
| | 1=iNPH | 0=no iNPH | -,00304* | ,00068 | ,000 | -,0047 | -,0014 |
| | | 2=AD | -,00148 | ,00068 | ,099 | -,0031 | ,0002 |
| | 2=AD | 0=no iNPH | -,00157 | ,00068 | ,071 | -,0032 | ,0001 |
| | | 1=iNPH | ,00148 | ,00068 | ,099 | -,0002 | ,0031 |
| Normalized_precuneus_right | 0=no iNPH | 1=iNPH | ,00139* | ,00036 | ,001 | ,0005 | ,0023 |
| | | 2=AD | ,00057 | ,00036 | ,359 | -,0003 | ,0015 |
| | 1=iNPH | 0=no iNPH | -,00139* | ,00036 | ,001 | -,0023 | -,0005 |

| | | | | | | | |
|---|---|---|---|---|---|---|---|
| | | 2=AD | -,00082 | ,00036 | ,078 | -,0017 | ,0001 |
| | 2=AD | 0=no iNPH | -,00057 | ,00036 | ,359 | -,0015 | ,0003 |
| | | 1=iNPH | ,00082 | ,00036 | ,078 | -,0001 | ,0017 |
| normalized_precuneus_left | 0=no iNPH | 1=iNPH | ,00166* | ,00034 | ,000 | ,0008 | ,0025 |
| | | 2=AD | ,00100* | ,00034 | ,012 | ,0002 | ,0018 |
| | 1=iNPH | 0=no iNPH | -,00166* | ,00034 | ,000 | -,0025 | -,0008 |
| | | 2=AD | -,00066 | ,00034 | ,168 | -,0015 | ,0002 |
| | 2=AD | 0=no iNPH | -,00100* | ,00034 | ,012 | -,0018 | -,0002 |
| | | 1=iNPH | ,00066 | ,00034 | ,168 | -,0002 | ,0015 |
| normiert_total_occipital_lobe | 0=no iNPH | 1=iNPH | ,00413* | ,00134 | ,007 | ,0009 | ,0074 |
| | | 2=AD | ,00171 | ,00134 | ,612 | -,0015 | ,0050 |
| | 1=iNPH | 0=no iNPH | -,00413* | ,00134 | ,007 | -,0074 | -,0009 |
| | | 2=AD | -,00242 | ,00134 | ,217 | -,0057 | ,0008 |
| | 2=AD | 0=no iNPH | -,00171 | ,00134 | ,612 | -,0050 | ,0015 |
| | | 1=iNPH | ,00242 | ,00134 | ,217 | -,0008 | ,0057 |
| normalized_occipital_right | 0=no iNPH | 1=iNPH | ,00166* | ,00067 | ,045 | ,0000 | ,0033 |
| | | 2=AD | ,00064 | ,00067 | 1,000 | -,0010 | ,0023 |
| | 1=iNPH | 0=no iNPH | -,00166* | ,00067 | ,045 | -,0033 | ,0000 |
| | | 2=AD | -,00102 | ,00067 | ,394 | -,0027 | ,0006 |
| | 2=AD | 0=no iNPH | -,00064 | ,00067 | 1,000 | -,0023 | ,0010 |
| | | 1=iNPH | ,00102 | ,00067 | ,394 | -,0006 | ,0027 |
| Normalized_occipital_left | 0=no iNPH | 1=iNPH | ,00247* | ,00074 | ,003 | ,0007 | ,0043 |
| | | 2=AD | ,00107 | ,00074 | ,453 | -,0007 | ,0029 |
| | 1=iNPH | 0=no iNPH | -,00247* | ,00074 | ,003 | -,0043 | -,0007 |
| | | 2=AD | -,00140 | ,00074 | ,184 | -,0032 | ,0004 |
| | 2=AD | 0=no iNPH | -,00107 | ,00074 | ,453 | -,0029 | ,0007 |
| | | 1=iNPH | ,00140 | ,00074 | ,184 | -,0004 | ,0032 |
| normalized_total_temporal_lobe | 0=no iNPH | 1=iNPH | ,01593* | ,00139 | ,000 | ,0126 | ,0193 |
| | | 2=AD | ,01728* | ,00139 | ,000 | ,0139 | ,0206 |
| | 1=iNPH | 0=no iNPH | -,01593* | ,00139 | ,000 | -,0193 | -,0126 |
| | | 2=AD | ,00135 | ,00139 | ,996 | -,0020 | ,0047 |
| | 2=AD | 0=no iNPH | -,01728* | ,00139 | ,000 | -,0206 | -,0139 |
| | | 1=iNPH | -,00135 | ,00139 | ,996 | -,0047 | ,0020 |
| normalized_temporal_right | 0=no iNPH | 1=iNPH | ,00839* | ,00087 | ,000 | ,0063 | ,0105 |
| | | 2=AD | ,00913* | ,00087 | ,000 | ,0070 | ,0113 |
| | 1=iNPH | 0=no iNPH | -,00839* | ,00087 | ,000 | -,0105 | -,0063 |
| | | 2=AD | ,00074 | ,00087 | 1,000 | -,0014 | ,0029 |
| | 2=AD | 0=no iNPH | -,00913* | ,00087 | ,000 | -,0113 | -,0070 |

| | | | | | | | |
|---|---|---|---|---|---|---|---|
| | | 1=iNPH | -,00074 | ,00087 | 1,000 | -,0029 | ,0014 |
| normalized _temporal_left | 0=no iNPH | 1=iNPH | ,00754* | ,00073 | ,000 | ,0058 | ,0093 |
| | | 2=AD | ,00814* | ,00073 | ,000 | ,0064 | ,0099 |
| | 1=iNPH | 0=no iNPH | -,00754* | ,00073 | ,000 | -,0093 | -,0058 |
| | | 2=AD | ,00061 | ,00073 | 1,000 | -,0012 | ,0024 |
| | 2=AD | 0=no iNPH | -,00814* | ,00073 | ,000 | -,0099 | -,0064 |
| | | 1=iNPH | -,00061 | ,00073 | 1,000 | -,0024 | ,0012 |
| normalized_total_Hippocampus | 0=no iNPH | 1=iNPH | ,00100 | ,00052 | ,169 | -,0003 | ,0023 |
| | | 2=AD | ,00041 | ,00052 | 1,000 | -,0008 | ,0017 |
| | 1=iNPH | 0=no iNPH | -,00100 | ,00052 | ,169 | -,0023 | ,0003 |
| | | 2=AD | -,00058 | ,00052 | ,783 | -,0018 | ,0007 |
| | 2=AD | 0=no iNPH | -,00041 | ,00052 | 1,000 | -,0017 | ,0008 |
| | | 1=iNPH | ,00058 | ,00052 | ,783 | -,0007 | ,0018 |
| normalized_Hippocampus_right | 0=no iNPH | 1=iNPH | ,00059 | ,00050 | ,721 | -,0006 | ,0018 |
| | | 2=AD | -,00003 | ,00050 | 1,000 | -,0013 | ,0012 |
| | 1=iNPH | 0=no iNPH | -,00059 | ,00050 | ,721 | -,0018 | ,0006 |
| | | 2=AD | -,00062 | ,00050 | ,657 | -,0018 | ,0006 |
| | 2=AD | 0=no iNPH | ,00003 | ,00050 | 1,000 | -,0012 | ,0013 |
| | | 1=iNPH | ,00062 | ,00050 | ,657 | -,0006 | ,0018 |
| normalized_Hippocampus_left | 0=no iNPH | 1=iNPH | ,00040* | ,00008 | ,000 | ,0002 | ,0006 |
| | | 2=AD | ,00044* | ,00008 | ,000 | ,0002 | ,0006 |
| | 1=iNPH | 0=no iNPH | -,00040* | ,00008 | ,000 | -,0006 | -,0002 |
| | | 2=AD | ,00004 | ,00008 | 1,000 | -,0002 | ,0002 |
| | 2=AD | 0=no iNPH | -,00044* | ,00008 | ,000 | -,0006 | -,0002 |
| | | 1=iNPH | -,00004 | ,00008 | 1,000 | -,0002 | ,0002 |
| normalized_total_Parahippocampus | 0=no iNPH | 1=iNPH | ,00111* | ,00017 | ,000 | ,0007 | ,0015 |
| | | 2=AD | ,00062* | ,00017 | ,001 | ,0002 | ,0010 |
| | 1=iNPH | 0=no iNPH | -,00111* | ,00017 | ,000 | -,0015 | -,0007 |
| | | 2=AD | -,00049* | ,00017 | ,011 | -,0009 | -,0001 |
| | 2=AD | 0=no iNPH | -,00062* | ,00017 | ,001 | -,0010 | -,0002 |
| | | 1=iNPH | ,00049* | ,00017 | ,011 | ,0001 | ,0009 |
| normalized_Gyrus_parahippocampalis_right | 0=no iNPH | 1=iNPH | ,00057* | ,00009 | ,000 | ,0004 | ,0008 |
| | | 2=AD | ,00033* | ,00009 | ,001 | ,0001 | ,0005 |
| | 1=iNPH | 0=no iNPH | -,00057* | ,00009 | ,000 | -,0008 | -,0004 |
| | | 2=AD | -,00024* | ,00009 | ,025 | -,0005 | ,0000 |
| | 2=AD | 0=no iNPH | -,00033* | ,00009 | ,001 | -,0005 | -,0001 |
| | | 1=iNPH | ,00024* | ,00009 | ,025 | ,0000 | ,0005 |
| | 0=no iNPH | 1=iNPH | ,00054* | ,00008 | ,000 | ,0003 | ,0007 |

| | | | | | | | |
|---|---|---|---|---|---|---|---|
| normalized_Gyrus_parahippocampalis_left | | 2=AD | ,00029* | ,00008 | ,003 | ,0001 | ,0005 |
| | 1=iNPH | 0=no iNPH | -,00054* | ,00008 | ,000 | -,0007 | -,0003 |
| | | 2=AD | -,00025* | ,00008 | ,010 | -,0005 | ,0000 |
| | 2=AD | 0=no iNPH | -,00029* | ,00008 | ,003 | -,0005 | -,0001 |
| | | 1=iNPH | ,00025* | ,00008 | ,010 | ,0000 | ,0005 |
| normalized_total_entorhinal | 0=no iNPH | 1=iNPH | ,00079* | ,00013 | ,000 | ,0005 | ,0011 |
| | | 2=AD | ,00091* | ,00013 | ,000 | ,0006 | ,0012 |
| | 1=iNPH | 0=no iNPH | -,00079* | ,00013 | ,000 | -,0011 | -,0005 |
| | | 2=AD | ,00012 | ,00013 | 1,000 | -,0002 | ,0004 |
| | 2=AD | 0=no iNPH | -,00091* | ,00013 | ,000 | -,0012 | -,0006 |
| | | 1=iNPH | -,00012 | ,00013 | 1,000 | -,0004 | ,0002 |
| normalized_Regio_entorhinalis_right | 0=no iNPH | 1=iNPH | ,00043* | ,00007 | ,000 | ,0003 | ,0006 |
| | | 2=AD | ,00050* | ,00007 | ,000 | ,0003 | ,0007 |
| | 1=iNPH | 0=no iNPH | -,00043* | ,00007 | ,000 | -,0006 | -,0003 |
| | | 2=AD | ,00006 | ,00007 | 1,000 | -,0001 | ,0002 |
| | 2=AD | 0=no iNPH | -,00050* | ,00007 | ,000 | -,0007 | -,0003 |
| | | 1=iNPH | -,00006 | ,00007 | 1,000 | -,0002 | ,0001 |
| normalized_Regio_entorhinalis_left | 0=no iNPH | 1=iNPH | ,00036* | ,00007 | ,000 | ,0002 | ,0005 |
| | | 2=AD | ,00042* | ,00007 | ,000 | ,0003 | ,0006 |
| | 1=iNPH | 0=no iNPH | -,00036* | ,00007 | ,000 | -,0005 | -,0002 |
| | | 2=AD | ,00006 | ,00007 | 1,000 | -,0001 | ,0002 |
| | 2=AD | 0=no iNPH | -,00042* | ,00007 | ,000 | -,0006 | -,0003 |
| | | 1=iNPH | -,00006 | ,00007 | 1,000 | -,0002 | ,0001 |
| normalized_total_Caudatus | 0=no iNPH | 1=iNPH | ,00011 | ,00031 | 1,000 | -,0006 | ,0009 |
| | | 2=AD | -,00015 | ,00031 | 1,000 | -,0009 | ,0006 |
| | 1=iNPH | 0=no iNPH | -,00011 | ,00031 | 1,000 | -,0009 | ,0006 |
| | | 2=AD | -,00026 | ,00031 | 1,000 | -,0010 | ,0005 |
| | 2=AD | 0=no iNPH | ,00015 | ,00031 | 1,000 | -,0006 | ,0009 |
| | | 1=iNPH | ,00026 | ,00031 | 1,000 | -,0005 | ,0010 |
| normalized_Nucleus_caudatus_right | 0=no iNPH | 1=iNPH | ,00008 | ,00017 | 1,000 | -,0003 | ,0005 |
| | | 2=AD | -,00005 | ,00017 | 1,000 | -,0005 | ,0003 |
| | 1=iNPH | 0=no iNPH | -,00008 | ,00017 | 1,000 | -,0005 | ,0003 |
| | | 2=AD | -,00013 | ,00017 | 1,000 | -,0005 | ,0003 |
| | 2=AD | 0=no iNPH | ,00005 | ,00017 | 1,000 | -,0003 | ,0005 |
| | | 1=iNPH | ,00013 | ,00017 | 1,000 | -,0003 | ,0005 |
| normalized_Nucleus_caudatus_left | 0=no iNPH | 1=iNPH | ,00003 | ,00015 | 1,000 | -,0003 | ,0004 |
| | | 2=AD | -,00010 | ,00015 | 1,000 | -,0005 | ,0003 |
| | 1=iNPH | 0=no iNPH | -,00003 | ,00015 | 1,000 | -,0004 | ,0003 |

| | | | | | | | |
|---|---|---|---|---|---|---|---|
| | | 2=AD | -,00013 | ,00015 | 1,000 | -,0005 | ,0002 |
| | 2=AD | 0=no iNPH | ,00010 | ,00015 | 1,000 | -,0003 | ,0005 |
| | | 1=iNPH | ,00013 | ,00015 | 1,000 | -,0002 | ,0005 |
| normalized_total_Putamen | 0=no iNPH | 1=iNPH | ,00012 | ,00006 | ,204 | ,0000 | ,0003 |
| | | 2=AD | -,00017* | ,00006 | ,025 | -,0003 | ,0000 |
| | 1=iNPH | 0=no iNPH | -,00012 | ,00006 | ,204 | -,0003 | ,0000 |
| | | 2=AD | -,00029* | ,00006 | ,000 | -,0004 | -,0001 |
| | 2=AD | 0=no iNPH | ,00017* | ,00006 | ,025 | ,0000 | ,0003 |
| | | 1=iNPH | ,00029* | ,00006 | ,000 | ,0001 | ,0004 |
| normalized_Putamen_right | 0=no iNPH | 1=iNPH | ,00037* | ,00011 | ,003 | ,0001 | ,0006 |
| | | 2=AD | -,00005 | ,00011 | 1,000 | -,0003 | ,0002 |
| | 1=iNPH | 0=no iNPH | -,00037* | ,00011 | ,003 | -,0006 | -,0001 |
| | | 2=AD | -,00042* | ,00011 | ,001 | -,0007 | -,0002 |
| | 2=AD | 0=no iNPH | ,00005 | ,00011 | 1,000 | -,0002 | ,0003 |
| | | 1=iNPH | ,00042* | ,00011 | ,001 | ,0002 | ,0007 |
| normalized_Putamen_left | 0=no iNPH | 1=iNPH | ,00049* | ,00013 | ,001 | ,0002 | ,0008 |
| | | 2=AD | -,00002 | ,00013 | 1,000 | -,0003 | ,0003 |
| | 1=iNPH | 0=no iNPH | -,00049* | ,00013 | ,001 | -,0008 | -,0002 |
| | | 2=AD | -,00051* | ,00013 | ,000 | -,0008 | -,0002 |
| | 2=AD | 0=no iNPH | ,00002 | ,00013 | 1,000 | -,0003 | ,0003 |
| | | 1=iNPH | ,00051* | ,00013 | ,000 | ,0002 | ,0008 |
| normalized_total_Pallidum | 0=no iNPH | 1=iNPH | ,00012 | ,00006 | ,204 | ,0000 | ,0003 |
| | | 2=AD | -,00017* | ,00006 | ,025 | -,0003 | ,0000 |
| | 1=iNPH | 0=no iNPH | -,00012 | ,00006 | ,204 | -,0003 | ,0000 |
| | | 2=AD | -,00029* | ,00006 | ,000 | -,0004 | -,0001 |
| | 2=AD | 0=no iNPH | ,00017* | ,00006 | ,025 | ,0000 | ,0003 |
| | | 1=iNPH | ,00029* | ,00006 | ,000 | ,0001 | ,0004 |
| normalized_Pallidum_right | 0=no iNPH | 1=iNPH | ,00008 | ,00003 | ,063 | ,0000 | ,0002 |
| | | 2=AD | -,00009* | ,00003 | ,020 | -,0002 | ,0000 |
| | 1=iNPH | 0=no iNPH | -,00008 | ,00003 | ,063 | -,0002 | ,0000 |
| | | 2=AD | -,00017* | ,00003 | ,000 | -,0003 | -,0001 |
| | 2=AD | 0=no iNPH | ,00009* | ,00003 | ,020 | ,0000 | ,0002 |
| | | 1=iNPH | ,00017* | ,00003 | ,000 | ,0001 | ,0003 |
| normalized_Pallidum_left | 0=no iNPH | 1=iNPH | ,00004 | ,00003 | ,723 | ,0000 | ,0001 |
| | | 2=AD | -,00008 | ,00003 | ,061 | -,0002 | ,0000 |
| | 1=iNPH | 0=no iNPH | -,00004 | ,00003 | ,723 | -,0001 | ,0000 |
| | | 2=AD | -,00012* | ,00003 | ,002 | -,0002 | ,0000 |
| | 2=AD | 0=no iNPH | ,00008 | ,00003 | ,061 | ,0000 | ,0002 |

| | | | | | | | |
|---|---|---|---|---|---|---|---|
| | | 1=iNPH | ,00012* | ,00003 | ,002 | ,0000 | ,0002 |
| normalized_total_Thalamus | 0=no iNPH | 1=iNPH | ,00193 | ,00128 | ,402 | -,0012 | ,0050 |
| | | 2=AD | -,00182 | ,00128 | ,473 | -,0049 | ,0013 |
| | 1=iNPH | 0=no iNPH | -,00193 | ,00128 | ,402 | -,0050 | ,0012 |
| | | 2=AD | -,00375* | ,00128 | ,012 | -,0068 | -,0006 |
| | 2=AD | 0=no iNPH | ,00182 | ,00128 | ,473 | -,0013 | ,0049 |
| | | 1=iNPH | ,00375* | ,00128 | ,012 | ,0006 | ,0068 |
| normalized_Thalamus_right | 0=no iNPH | 1=iNPH | ,00084* | ,00016 | ,000 | ,0004 | ,0012 |
| | | 2=AD | -,00024 | ,00016 | ,450 | -,0006 | ,0002 |
| | 1=iNPH | 0=no iNPH | -,00084* | ,00016 | ,000 | -,0012 | -,0004 |
| | | 2=AD | -,00108* | ,00016 | ,000 | -,0015 | -,0007 |
| | 2=AD | 0=no iNPH | ,00024 | ,00016 | ,450 | -,0002 | ,0006 |
| | | 1=iNPH | ,00108* | ,00016 | ,000 | ,0007 | ,0015 |
| normalized_Thalamus_left | 0=no iNPH | 1=iNPH | ,00109 | ,00124 | 1,000 | -,0019 | ,0041 |
| | | 2=AD | -,00158 | ,00124 | ,618 | -,0046 | ,0014 |
| | 1=iNPH | 0=no iNPH | -,00109 | ,00124 | 1,000 | -,0041 | ,0019 |
| | | 2=AD | -,00267 | ,00124 | ,102 | -,0057 | ,0004 |
| | 2=AD | 0=no iNPH | ,00158 | ,00124 | ,618 | -,0014 | ,0046 |
| | | 1=iNPH | ,00267 | ,00124 | ,102 | -,0004 | ,0057 |
| normalized_brainstem | 0=no iNPH | 1=iNPH | ,00154* | ,00044 | ,002 | ,0005 | ,0026 |
| | | 2=AD | -,00207* | ,00044 | ,000 | -,0031 | -,0010 |
| | 1=iNPH | 0=no iNPH | -,00154* | ,00044 | ,002 | -,0026 | -,0005 |
| | | 2=AD | -,00361* | ,00044 | ,000 | -,0047 | -,0025 |
| | 2=AD | 0=no iNPH | ,00207* | ,00044 | ,000 | ,0010 | ,0031 |
| | | 1=iNPH | ,00361* | ,00044 | ,000 | ,0025 | ,0047 |
| normalized_mesencephalon | 0=no iNPH | 1=iNPH | ,00042 | ,00041 | ,924 | -,0006 | ,0014 |
| | | 2=AD | -,00111* | ,00041 | ,023 | -,0021 | -,0001 |
| | 1=iNPH | 0=no iNPH | -,00042 | ,00041 | ,924 | -,0014 | ,0006 |
| | | 2=AD | -,00153* | ,00041 | ,001 | -,0025 | -,0005 |
| | 2=AD | 0=no iNPH | ,00111* | ,00041 | ,023 | ,0001 | ,0021 |
| | | 1=iNPH | ,00153* | ,00041 | ,001 | ,0005 | ,0025 |
| normalized_Pons | 0=no iNPH | 1=iNPH | ,00128 | ,00053 | ,055 | ,0000 | ,0026 |
| | | 2=AD | -,00021 | ,00053 | 1,000 | -,0015 | ,0011 |
| | 1=iNPH | 0=no iNPH | -,00128 | ,00053 | ,055 | -,0026 | ,0000 |
| | | 2=AD | -,00148* | ,00053 | ,019 | -,0028 | -,0002 |
| | 2=AD | 0=no iNPH | ,00021 | ,00053 | 1,000 | -,0011 | ,0015 |
| | | 1=iNPH | ,00148* | ,00053 | ,019 | ,0002 | ,0028 |
| normalized_Cerebellum | 0=no iNPH | 1=iNPH | ,01068* | ,00241 | ,000 | ,0048 | ,0165 |

| | | | | | | | |
|---|---|---|---|---|---|---|---|
| | | 2=AD | ,00004 | ,00241 | 1,000 | -,0058 | ,0059 |
| | 1=iNPH | 0=no iNPH | -,01068* | ,00241 | ,000 | -,0165 | -,0048 |
| | | 2=AD | -,01063* | ,00241 | ,000 | -,0165 | -,0048 |
| | 2=AD | 0=no iNPH | -,00004 | ,00241 | 1,000 | -,0059 | ,0058 |
| | | 1=iNPH | ,01063* | ,00241 | ,000 | ,0048 | ,0165 |
